# Identification of potential serum biomarkers of acute paraquat poisoning in humans using an iTRAQ quantitative proteomic†

**DOI:** 10.1039/c7ra12956d

**Published:** 2018-03-16

**Authors:** Liming Wei, Yi Wang, Ling Lin, Lei Zhang, Yan Shi, Ping Xiang, Shujun Cao, Min Shen, Pengyuan Yang

**Affiliations:** Shanghai Key Laboratory of Forensic Medicine, Shanghai Forensic Service Platform, Institute of Forensic Science, Ministry of Justice Shanghai China shenm@ssfjd.cn; Institutes of Biomedical Sciences & Department of Chemistry, Fudan University Shanghai China pyyang@fudan.edu.cn; Shanghai Songjiang District Central Hospital Shanghai China shujun-cao@163.com

## Abstract

Paraquat (PQ) poisoning has high mortality rates in many countries. Due to it readily being absorbed through the gastrointestinal tract and rapidly excreted in the urine, few biomarkers possess satisfactory specificity and sensitivity in diagnostic and forensic practices. To investigate serum biomarkers in patients with PQ poisoning, pooled sera was analyzed using a proteomic approach based on iTRAQ coupled LC-MS/MS. Of the 413 proteins identified with high confidence, 81 were found to be differentially expressed (1.5-fold change) in the sera of patients with PQ poisoning. The differential expression pattern of 4 of these proteins was validated by enzyme-linked immunosorbent assay (ELISA) in clinical samples. A sera sample from a PQ poisoning patient has shown relatively increased abundance of S100A8 and S100A9. The overexpression of S100A8 and S100A9 was further validated in the lung tissue of PQ-treated rat associated with lung damage. Meanwhile, we identified another two down-expressed proteins, transferrin receptor protein 1 (TfR1) and serum amyloid P-component (SAP), which may be also practicable in human clinical samples as PQ poisoning serum biomarkers. Furthermore, receiver operating characteristic curve analysis confirmed that the expression levels of S100 alarmins, TfR1 and SAP in patient serum could provide a discriminatory diagnostic test for predicting PQ poisoning in patients. Therefore, our results suggest that increased serum levels of S100 alarmins and decreased serum levels of TfR1 and SAP may constitute potential biomarkers for the prediction of PQ poisoning in humans, and might be novel therapeutic targets in PQ poisoning.

## Abstract 2

Using an iTRAQ quantitative proteomic, S100 alarmins, TfR1 and SAP have been discovered as potential indicators to paraquat poisoning in humans.

## Graphical abstract

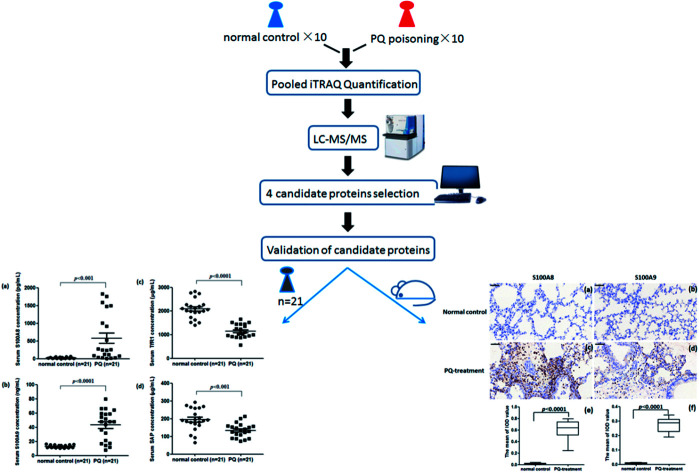

## 1. Introduction

Paraquat dichloride (1,1′-dimethyl-4,4′-bipyridinium dichloride methyl viologen, PQ) is one of the most widely used herbicides in the world and can be lethal when ingested by humans.^1,2^ With the widespread use of this herbicide in many countries, the incidence of PQ poisoning has trended upward over the last several decades due to accidental and intentional ingestion of the product. Although PQ is absorbed into the bloodstream through the gastrointestinal tract, the lungs are the primary site of PQ-induced injury in humans and animals. It is well-established that PQ poisoning can cause acute lung injury and pulmonary fibrosis culminating in reduced functional capacity, which is the most common cause of death in patients with PQ poisoning.^3,4^ Although researchers tried to seek efficacious therapeutic modalities for the management of PQ poisoning, the treatment of severe PQ poisoning is less effective and mortality rates remain high with a mortality rate of nearly 80%.^5^

In the body, PQ is readily absorbed through the gastrointestinal tract and rapidly excreted in the urine.^6,7^ Prediction of survival in patients with PQ intoxication is closely related to the amount of PQ ingested, as well as the amount of time that passes between exposure and treatment. However, there is a lack of effective diagnostic methods for PQ intoxication due to considerable clearance of the compound from the blood within hours following exposure. Furthermore, many hospitals do not have the facilities to measure serum PQ levels, so the practical value of using serum levels of this compound as a diagnostic marker is limited. At present, several novel methods and indicators have been reported to evaluate the diagnosis of PQ intoxication, which including arterial blood gas analysis,^8^ serum creatinine or cystatin C (ref. 9), serum uric acid and pentraxin-3 (ref. 10 and 11) and urinary neutrophil gelatinase-associated lipocalin (NGAL)^12^ among others. However, a major limitation of these indicators is the unreliability and instability in a number of acutely poisoned patients. Thus, it is essential to investigate and identify diagnostic biomarkers for PQ intoxication. Since the mechanism of PQ toxicity is not yet fully understood, studies focusing on identifying the mechanisms of toxicity, as well as biomarkers of PQ-induced pulmonary fibrosis, will be essential for developing treatments and reducing mortality associated with PQ poisoning.

In the postgenomic area, proteomics provides an effective approach to investigate all the proteins expressed by a genome in a special time and physiological or pathological condition. Furthermore, proteomics has become a direct and fast way for biomarker discovery, which will lead to new diagnostic and therapeutic targets in a variety of sample types.^13,14^ Therefore, proteomics has been widely applied in the research of disease pathogenesis, clinical diagnosis and treatment. The isobaric tags for relative and absolute quantitation (iTRAQ) technology provides a means for relative quantification of proteins from multiple specimens (up to eight) within a single MS analysis. Isobaric tagging is achieved by using chemical moieties or tags which are identical in mass, so that all labelled peptides are isobaric, chromatographically indistinguishable and yielding a single peak in the mass spectrum for both samples (for instance, treated and non-treated samples). Then, the relative abundances of the isobarically tagged peptides are revealed when the moieties fragment during MS/MS experiments to release reporter ions with different masses, which can be correlated to a particular sample source.^15^

Here, we examined the differential expression of proteins in pooled serum samples from patients with PQ intoxication using iTRAQ-based quantitative proteomics. Of the 81 differentially expressed proteins identified in pooled serum from patients with PQ poisoning, S100A8 and S100A9 were found to be increased more than 1.8-fold and 3.0-fold, respectively. ELISA was used to validate the differential expression patterns of 4 candidate biomarkers, including protein S100-A8 (S100A8), protein S100-A9 (S100A9), transferrin receptor protein 1 (TfR1), and serum amyloid P-component (SAP), in serum from PQ poisoning patients. Meanwhile, to evaluate the relationship of S100A8 and S100A9 with PQ-induced pulmonary fibrosis, immunohistochemical (IHC) analysis confirmed the increased expression of these proteins in lung tissue from a rat model of PQ poisoning. Furthermore, the serum concentrations of TfR1 and SAP were found to be inversely correlated with PQ poisoning. The prognostic value of serum TfR1 and SAP concentrations, as well as their possible therapeutic applications, may also be worth further investigation.

## 2. Material and methods

### 2.1 Sample collection

#### 2.1.1 Serum sample collection

All human serum sample and rat sample in this study were approved by the Ethics Committee of Institute of Biomedical Sciences, Fudan University and Institute of Forensic Science, Ministry of Justice, respectively. Informed consent was obtained from all participants. All the methods were carried out in accordance with the NIH guidelines. 21 consecutive patients who presented to the Department of Emergency Medicine at the Ruijin Hospital and Shanghai Sixth People's Hospital from May 2013 to February 2014 with acute PQ poisoning were enrolled in the study (ESI Table S1†). 21 outpatients were recruited from the outpatient department to serve as normal controls. Blood samples from patients with PQ poisoning and normal controls were obtained *via* vein blood sampling. Immediately after collection, the sera were centrifuged at 3000 rpm for 20 min. The serum was stored at −80 °C until analysis.

#### 2.1.2 Animal model

A total of 12 adult male rat weighing 100–150 g were purchased from Shanghai Laboratory Animal Center, Chinese Academy of Sciences. The rat were randomly divided into two groups (*n* = 6 for each group). The experimental group received consecutive oral injections of PQ (10 mg kg^−1^) every six days, while the control group received an equivalent volume of sterile saline. The dosage of the PQ was following the former report with minor revision.^16^ The rats were then sacrificed by cervical dislocation after 21 days. All animals were used in accordance with rules published by the National Institutes of Health. The Institute of Forensic Science, Ministry of Justice Animal Use and Care Committee approved the rat procedures.

### 2.2 Depletion of high-abundance serum proteins and iTRAQ labeling

100 μL of serum samples from 10 patients with PQ poisoning were pooled for iTRAQ analysis. The pooled serum samples from PQ poisoning patients and healthy control were subjected to high abundance protein depletion using ProteoMiner™ protein enrichment kits (Bio-Rad Laboratories, Inc., USA) according to the manufacturer's instructions. Protein concentration was determined using the 2-D quantification kit (GE Healthcare Bio-Sciences, USA).

After abundant protein depletion and concentration measurement, a total of 100 μg of each sample was reduced and alkylated according to the kit protocol (Applied Biosystems Inc., Foster City, CA). Then protein solutions were digested overnight at 37 °C with sequence grade modified trypsin (Promega) (1 : 50). After trypsin digestion, iTRAQ regents (113, 114) in 70 μL isopropanol were added separately into each tube and incubated at room temperature for 1 h. The labeled peptide solutions were then pooled and dried by vacuum centrifugation.

### 2.3 Off-line 2D LC-MS analysis

The lyophilized iTRAQ-labeled peptide sample was desalted and re-dissolved in solvent A (20 mM ammonium formate in water, pH 10.0, which was adjusted with ammonium hydroxide), and then the mixture was fractionated by reverse phase chromatography at high pH (high pH RPLC) using Acquity UPLC (Waters Corporation, Milford, MA) connected to a reverse phase column (XBridge C18, 3.5 μm, 2.1 mm × 150 mm, Waters Corporation, USA). High pH separation was performed using a linear gradient starting from 5% solvent B to 35% solvent B in 45 min (solvent B: 20 mM ammonium formate in 90% ACN, pH 10.0, which was adjusted with ammonium hydroxide). The column flow rate was maintained at 200 μL min^−1^ and column temperature was maintained at room temperature. Finally 15 fractions were collected, and each fraction was dried in a vacuum concentrator for the next step.

The fractions were re-suspended with 25 μL solvent C (solvent C: 0.1% formic acid in 5% ACN aqueous solution) and then were separated and analyzed by on-line nano LC-electrospray tandem mass spectrometry. The experiments were accomplished on a Nano-Acquity UPLC system (Waters Corporation, USA) connected to a quadrupole-Orbitrap mass spectrometer (LTQ-Orbitrap) (Thermo Finnigan, San Jose, CA, USA). 8 μL of peptide fraction was loaded onto the trap column (Thermo Scientific Acclaim PepMap C18, 100 μm × 2 cm) with a flow rate of 10 μL min^−1^ for 3 min, and subsequently was separated on the analytical column (Acclaim PepMap C18, 75 μm × 50 cm) with a linear gradient, from 5% to 45% solvent D (solvent D: ACN including 0.1% formic acid) in 120 min. The column flow rate was maintained at 300 nL min^−1^. An electrospray voltage of 2.5 kV *versus* the inlet of the mass spectrometer was used.

The LTQ-Orbitrap was operated in information-dependent data acquisition mode to switch automatically between MS and MS/MS acquisition. Mass spectra were acquired across the mass range of 350–1250 *m*/*z* using 250 ms accumulation time per spectrum. LTQ-Orbitrap survey scans were collected at a resolution of 60 000 (at *m*/*z* 200) while a resolution of 13 500 (at *m*/*z* 200) was used for the collection of tandem mass spectra. Tandem mass spectra were acquired over the mass range 100–1250 *m*/*z* in high sensitivity mode with rolling collision energy. The 15 most intense precursors were selected for fragmentation per cycle with dynamic exclusion time of 40 s. The normalized collision energy was 30 eV and the underfill ratio was defined as 0.1% on the LTQ-Orbitrap.

### 2.4 Data analysis

#### 2.4.1 Identification and quantification of proteins

Protein identification was carried out using the MASCOT search engine (version 2.2.1; Matrix Science, London, UK) embedded into Proteome Discoverer 1.3 (Thermo Electron, San Jose, CA, USA), searching against the Uniprot database of human protein sequences (03–2015, 20 199 entries, downloaded from: http://www.uniprot.org/) and the decoy database. Search parameters were set as follows: monoisotopic mass, peptide mass tolerance at ±10 ppm and fragment mass tolerance at 0.05 Da, trypsin as the enzyme and allowing up to two missed cleavages. Variable modifications were defined as oxidation of methionine and iTRAQ 8-plex labeling tyrosine, while lysine and N-term of peptides labeled by iTRAQ 8-plex and carbamidomethylation of cysteine were specified as fixed modifications. Scaffold software (version Scaffold_4.3.2, Proteome Software Inc., Portland, OR) was used to supervise the identification quality. The identification of proteins was accepted when the false discovery rate (FDR) of both proteins and peptides identification as set to be less than 0.01. Protein identification was supported by at least one unique peptide as former report.^17^

#### 2.4.2 Ingenuity pathway analysis

The iTRAQ results were evaluated using ingenuity pathway analysis (IPA; Ingenuity Systems, Mountain View, CA; www.ingenuity.com). This software analyses protein expression data in the context of known biological response and regulatory networks, as well as other higher-order response pathways. The data set that contained the differentially expressed proteins identified in the iTRAQ experiment was converted by IPA to ‘fold change’ and uploaded into IPA. No expression value cutoff was selected, and we were interested in looking at both up- and down-regulated proteins. Hypothetical networks were generated from these proteins and other proteins from the database that were needed to fill out a protein cluster. Network generation was optimized for the inclusion of as many proteins from the input expression profile as possible and aimed for highly connected networks. IPA computes a score for each possible network according to the fit of that network to the input proteins. The score is calculated as the negative base-10 logarithm of the *p*-value that indicates the probability of the input proteins in a given network being found together as a result of random chance. Consequently, scores of 2 or higher have at least a 99% confidence of not being generated by random chance alone.

### 2.5 Serum S100A8, S100A9, TfR1, SAP validation by ELISA

The concentrations of S100A8 (ThermoFisher Scientific, EHS100A8, USA), S100A9 (Abnova, KA1050, USA), TfR1 (R&D systems Inc, DY2474, USA), and SAP (Abcam, ab137970, USA) in serum from 21 patients and 21 health controls were quantified using ELISA according to the manufacturer's instructions. Meanwhile, the serum concentrations of S100A8 (Abcam, ab213886, USA) and S100A9 (Abcam, ab213887, USA) were detected in rat model. All assays were performed in duplicate.

### 2.6 Histological analysis

To quantify and determine the location of S100A8 and S100A9 in lung tissue from rat treated with PQ or saline, we also performed histological and immunohistochemical analyses.

After sacrificing the rat, the lung were removed and lung tissues were fixed with 4% formalin, embedded in paraffin, and cut into 5 mm-thick sections for histological analysis. For hematoxylin and eosin (H&E) and Masson's trichrome staining, following deparaffinization, the sections were stained with an H&E kit or a Masson's trichrome staining kit (Biyuntian, Inc., Nantong, China) according to the manufacturer instructions, respectively. The slides with H&E staining and Masson's trichrome staining were examined using light microscopy (Leica, Germany) and with the aid of a software image analyser (Video Test-Master 4.0 software).

Immunohistochemical staining for S100A8 and S100A9 were performed on transverse sections of the lung tissue. Sections were de-waxed in xylol, rehydrated in decreasing concentrations of alcohol, and then subjected to microwave antigen retrieval (10 min in 0.1 M citrate acid buffer solution, pH 6). Sections were blocked in 0.3% hydrogen peroxide and PBS containing 10% fetal calf serum (FBS) and 0.1% Triton X-100. Sections were then incubated with rabbit antibodies against S100A8 (1 : 100) (Abcam ab92331) and S100A9 (1 : 200) (Abcam ab105472) in 10% FBS overnight at 4 °C. After washing, the sections were incubated with peroxidase-conjugated goat anti-rabbit IgG (Abcam) in 10% FBS containing 0.1% Triton X-100 for 20 min at room temperature and colored using diaminobenzidine (DAB). Images were taken with a microscope (Nikon, Japan). Image-pro Plus 6.0 software was used to calculate stained area and Integrated Optical Density (IOD). The average optical density (mean density) represented the intensity of protein expression and was counted in 4 random fields (×40 objective) per section. The mean density is equal to (IOD SUM)/area. For exact analysis, three sections were prepared at similar plane for each sample.

### 2.7 Statistical analysis

Statistical analysis was performed using GraphPad Prism software (GraphPad software, San Diego, CA). Differences between two groups were assessed by Student's *t* test. Significance was defined as *p* < 0.05.

## 3. Results

### 3.1 Analysis of proteomics changes in the sera of human patients with PQ poisoning

**Fig. 1:**
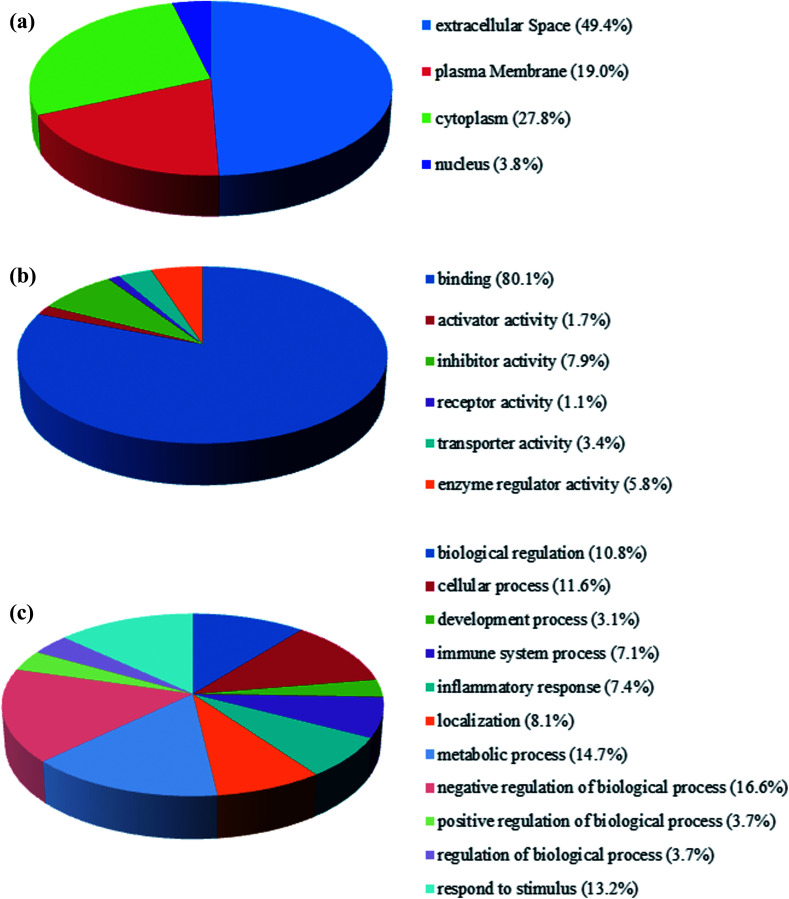
GO annotation and functional classification of proteins differentially expressed in the serum of patients with PQ poisoning. GO terms for subcellular location distribution (a), molecular functions (b) and biological process (c).

The fraction of serum samples from patients with PQ poisoning, as well as that from healthy controls, obtained after the depletion of high-abundance proteins, was labeled with iTRAQ and analyzed *via* LC-MS/MS with two technical replicates per sample (ESI Fig. S1†). In the combination of the two technical replicates, a total of 413 unique proteins containing at least one unique peptide were identified with confidence values ≥99% of FDR estimation ≤1% (ESI Table S2†). 283 of them were detected in both independent experiments (ESI Table S3†). In the first experiment, 143 proteins were found to be changed for more than 1.5-fold, including 48 proteins up-regulated and 36 proteins down-regulated. In the second, 122 proteins were changed, 69 proteins up-regulated and 53 down-regulated. Combination of two independent experiments showed 81 identified proteins including 30 up-regulated proteins and 51 down-regulated respectively, in the serum samples of patients with PQ intoxication based on fold change (fold change > 1.5 or ≤ 0.667) (*e.g.* the standard deviation distribution of relative changes in protein levels with the *p*-value < 0.05 (ESI Table S4†) are shown in volcano plots in ESI Fig. S2†). To gain insight into the biological functions of the proteins exhibiting altered expression in the patients with PQ poisoning, the differentially expressed proteins were categorized according to their annotation in the Gene Ontology (GO) database. The subcellular distributions that were enriched for the 81 differentially expressed proteins were the extracellular space (49.4%), plasma membrane (19.0%), cytoplasm (17.8%) and partly in the nucleus, which implies that most of the differentially expressed proteins are secreted proteins (Fig. 1a). According to the molecular function classifications, the majority of the differentially expressed proteins are associated with binding activity (80.1%), inhibitor activity (7.9%), enzyme regulator activity (5.8%), and transport activity (3.4%) (Fig. 1b). Furthermore, the differentially expressed proteins were more frequently involved in biological regulation, cellular processes, processes associated with response to stimuli, regulation of biological processes, metabolic processes, and immune system processes (Fig. 1c).

**Fig. 2:**
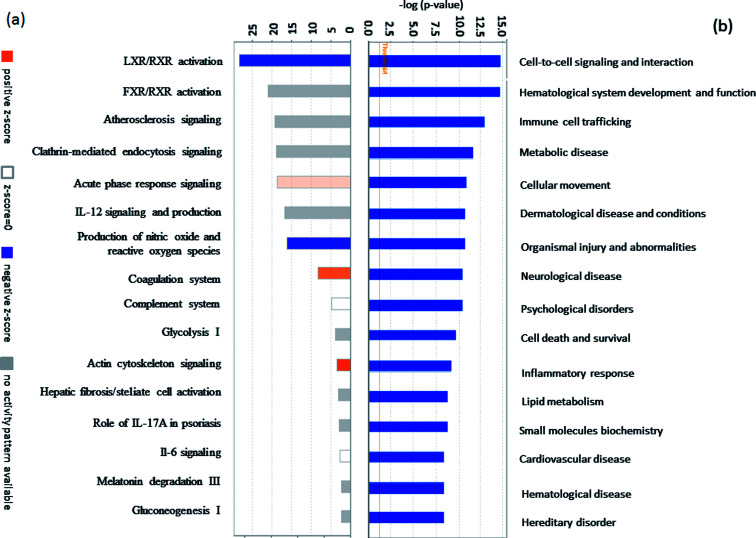
(a) The vertical axis shows the pathway name and the horizontal axis is the *p*-value of each pathway. (b) The vertical axis shows the name of each disease and function, and the horizontal axis is the *p*-value of each disease and function.

To identify potential pathways and/or interaction networks affected by PQ poisoning, the 81 differentially expressed proteins were further analyzed using Ingenuity Pathway Analysis (IPA) (Ingenuity Systems, Redwood City, CA) software. The identified pathways associated with the differentially expressed proteins are summarized in Table S5,† and the top 16 pathways are shown in Fig. 2a. IPA analysis identified pathways related to inflammation and immunity, including LXR/RXR activation, FXR/RXR activation, clathrin-mediated endocytosis signaling, acute phase response signaling, IL-12 signaling, and production of nitric oxide and reactive oxygen species. Pathways related to movement and injury, such as actin cytoskeleton signaling, hepatic fibrosis/stellate cell activation, and integrin signaling were also identified by IPA analysis. S100 family proteins, TPM, and APOE family proteins were identified among the top hit list in PQ poisoning group. The potential relationship of these differentially expressed depression-associated proteins with disease and function is shown in Fig. 2b. In particular, these differentially expressed proteins are associated with immune cell trafficking, inflammatory response, organismal injury and abnormalities, and metabolic disease. S100A8 and S100A9 were identified as the potentially perturbed proteins, which were related to immunological disease and metabolic disease.

**Fig. 3:**
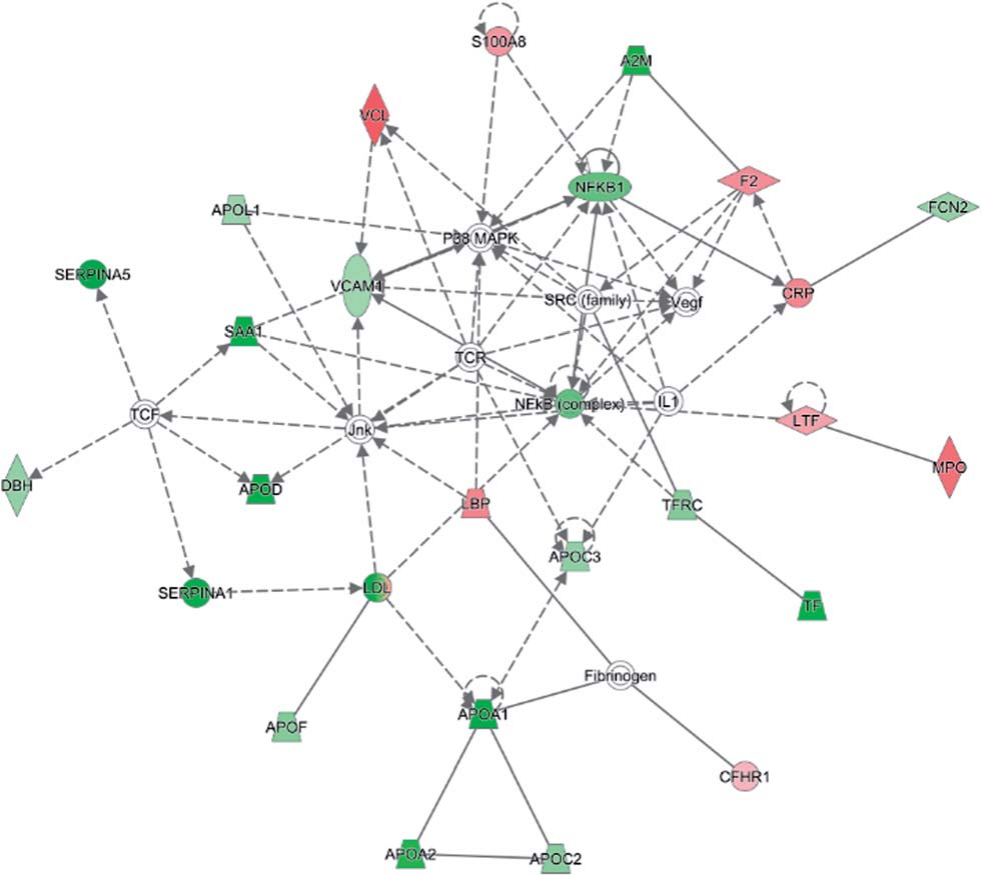
Twenty-five proteins showed a significant change in expression between the two subjects and were found to function together in a network revealed altered regulation of the cellular movement, hematological system development and function, immune cell trafficking. Coloring is based on gene expression values, down-regulation is depicted in green and up-regulation is depicted in red. Genes with no coloring were added from the ingenuity knowledge database. Solid lines correspond to indirect interactions.

To investigate the interaction of the differentially expressed proteins, we searched for the IPA network database. In this work, IPA generated several networks illustrated strong hubs of dysregulation in PQ poisoning patients. The highest scoring IPA network contained 35 focus molecules and had a significance score of 50. The network revealed altered regulation of the cellular movement, hematological system development and function, lipid metabolism, which includes hubs of activity in the nuclear factor kappa B subunit 1 (NFKB1), vascular cell adhesion molecule 1 (VACM1), and the P38 mitogen-activated protein kinase (P38 MAPK) pathways (Fig. 3). Especially, S100A8 and TfR1 were identified in the same pathway. Based on this network, 25 proteins were found to be differentially expressed in PQ poisoning patients compared with healthy controls. Among these, 8 proteins displayed increased expression, and 17 proteins exhibited decreased expression, as shown in Table S6.† Almost 48% proteins are related as potential diagnosis molecules in disease.

### 3.2 ELISA validation of differentially expressed proteins in PQ poisoning serum

**Fig. 4:**
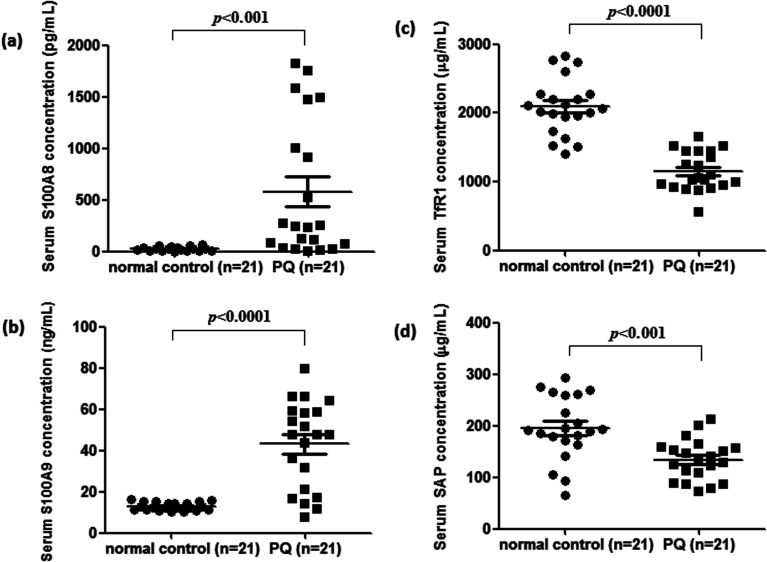
Serum expression of S100A8 (a), S100A9 (b), TfR1 (c), and SAP (d) detected by ELISA in the different clinical groups, respectively. Increased S100A8 and S100A9 serum levels were observed for all PQ poisoning patients (*n* = 21) compared to healthy controls (*n* = 21). Conversely, PQ poisoning groups showed significantly lower serum levels of TfR1 and SAP than the healthy group.

**Fig. 5:**
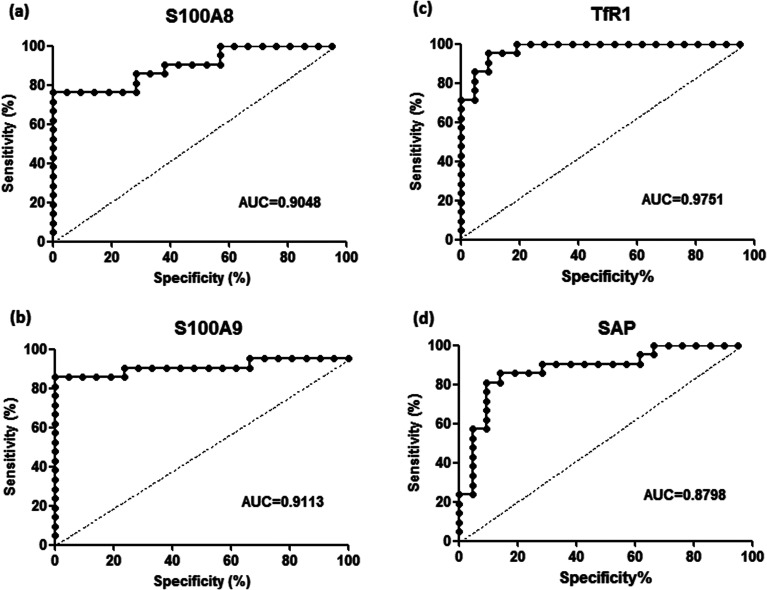
Receiver-operating characteristic (ROC) analysis for S100A8 (a), S100A9 (b), TfR1 (c), and SAP (d) to discriminate PQ poisoning patients from healthy controls, respectively.

To validate the differentially expressed proteins determined by iTRAQ-LC-MS/MS, ELISA analysis was utilized to analyze four candidate proteins, S100A8, S100A9, TfR1, and SAP, in the serum samples of 21 patients with PQ poisoning. These proteins were selected on the basis of several factors, including large fold changes in expression, published literature indicating an association with hematological system development and function, immune cell trafficking, response to stimulus, potential secreted proteins or cytoplasmic localization, and the availability of commercial antibodies. The represent MS spectra of these four proteins were shown in ESI Fig. S3–S6.† The results of our ELISA analysis confirmed the findings obtained using iTRAQ-based proteomics, highlighting the credibility of the proteomic analysis. Compared with the healthy control group (*n* = 21), the PQ poisoning patient group (*n* = 21) showed significantly elevated serum levels of S100A8 (normal 22.8 ± 4.7 pg mL^−1^*versus* PQ 577.2 ± 144.5 pg mL^−1^, *p* = 0.0004) and S100A9 (normal 13.0 ± 0.4 ng mL^−1^*versus* PQ 43.12 ± 4.61 ng mL^−1^, *p* < 0.0001) (Fig. 4a and b). The serum levels of the other two proteins were significantly lower in PQ poisoning patients than in healthy controls (TfR1 normal 2091.0 ± 88.7 μg mL^−1^*versus* PQ 1154.0 ± 61.6 μg mL^−1^, *p* < 0.0001; SAP normal 195.8 ± 13.5 μg mL^−1^*versus* PQ 134.3 ± 8.4 μg mL^−1^, *p* = 0.0004) (Fig. 4c and d). These findings indicate that these candidate proteins might be potential serum biomarkers for PQ poisoning in patients. To further evaluate the diagnostic significance of serum S100A8 and S100A9 expression, we constructed a receiver operation characteristic (ROC) curve by plotting sensitivity *versus* specificity (Fig. 5). The area under the ROC curve (AUC), a commonly used indicator for estimating the diagnostic efficacy of a potential biomarker, was subsequently calculated. For differentiating patients with PQ poisoning from healthy controls, the AUC was determined to be 0.9048 (95% confidence interval, 0.8067–0.9938) for S100A8 and 0.9113 (95% confidence interval, 0.7999–1.019) for S100A9 (Fig. 5a and b). When a cutoff value of 64.13 pg mL^−1^ was set for S100A8, the sensitivity and specificity for differentiating PQ poisoning from healthy controls were 76.2% and 95.2%, respectively. At a cutoff value of 16.02 ng mL^−1^, S100A9 had a sensitivity of 85.7% and specificity of 95.2% in detecting PQ poisoning. Even though the expression of TfR1 and SAP were inversely correlated with PQ poisoning, they may also have potential as diagnostic markers for PQ poisoning. The diagnostic values of serum TfR1 and SAP were evaluated by ROC curve analysis. The AUC was 0.9751 (95% confidence interval, 0.9378–1.012) for TfR1 and 0.8798 (95% confidence interval, 0.7711–0.9885) for SAP (Fig. 5c and d).

### 3.3 Verification of S100A8 and S100A9 highly associated with lung damage and pulmonary fibrosis in a rat model of PQ-induced toxicity

**Fig. 6:**
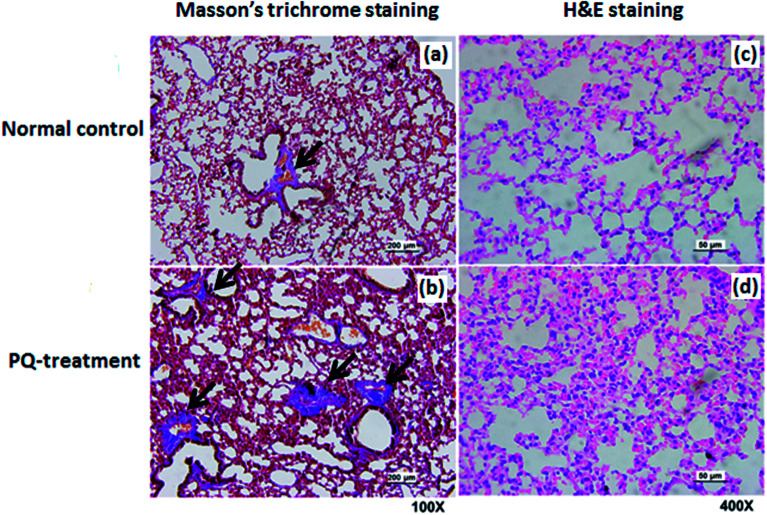
Histological analysis of lung tissue from a rat model of PQ-induced toxicity and healthy controls. Photomicrograph of normal lung tissue stained with Masson's trichrome staining, 100× (a) and H&E staining, 400× (c), respectively. Photomicrograph of lung tissue from rat treated with PQ stained with Masson's trichrome staining, 100× (b) and H&E staining, 400× (d), respectively.

**Fig. 7:**
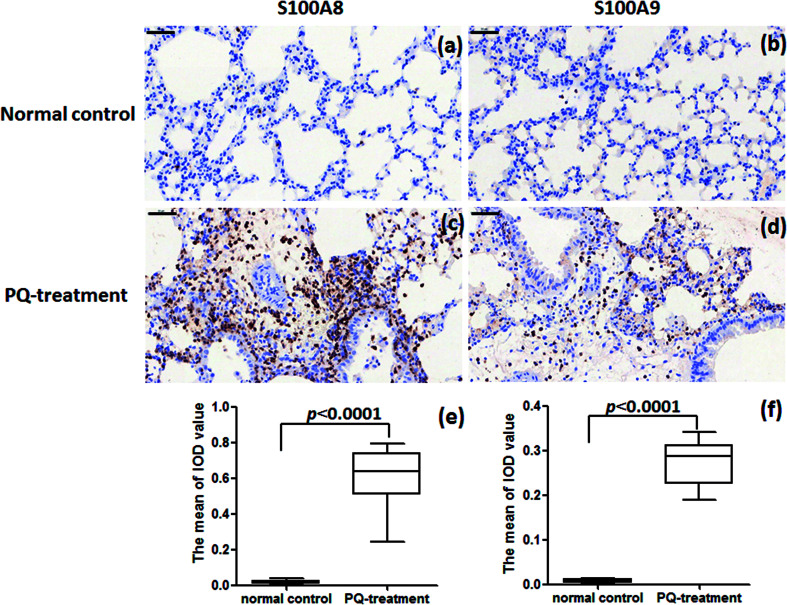
IHC analysis of S100A8 and S100A9 expression in lung tissue from PQ-treated rat and healthy controls. (a) and (b) Absence of staining for S100A8 and S100A9 in the normal lung tissue, respectively. (c) and (d) Marked S100A8 and S100A9 staining in lung tissue from rat treated with PQ. (Original magnification, 40×. Scale bars = 50 μm). Results of S100A8 (e) and S100A9 (f) immunohistochemistry in the cytoplasm and cell membrane in the lung tissues were quantitated by average optical of positive staining per 200 field.

For further assessment in terms of S100A8 and S100A9 relevance in PQ poisoning, a rat model of PQ-induced toxicity was prepared by treating rat with PQ for 21 days. Histopathological and IHC analyses were carried out to investigate PQ-induced pulmonary fibrosis in PQ-treated rat. To investigate the effects of PQ on lung tissue, lung sections from PQ-treated rat were stained with Masson's trichrome and standard haematoxylin & eosin (H&E) (Fig. 6). No signs of lung damage were observed in representative histological sections from control animals (Fig. 6a). By contrast, histopathological examination of lung tissue from PQ-treated rat showed significant alterations in tissue structure (Fig. 6b). H&E staining indicated increased thickness of alveolar septa due to inflammation. Masson's trichrome staining revealed an increase in collagen deposition (blue) in the lung tissue of rat treated with PQ compared with that in control lung tissue, indicating pulmonary interstitial fibrosis in PQ-treated rat. IHC was employed to further investigate the expression of S100A8 and S100A9 in PQ-treated rat lung tissue. The specific sites of positive staining for S100A8 and S100A9 were mainly localized to the cytoplasm and cell membrane in the PQ-treated lung tissues, whereas no staining or only faint staining was found in tissue from the control group (Fig. 7). The integrated optical density (IOD) values of S100A8 and S100A9 are presented in the Fig. 7e and f, respectively. Using the Spearman's rank correlation coefficient, strong S100A8 and S100A9 were detected in PQ-treated lung tissues (IOD 0.60 ± 0.056 and IOD 0.27 ± 0.017, *n* = 6), which were significantly higher than that in normal ones (IOD 0.018 ± 0.0035 and 0.0067 ± 0.0012, *n* = 6) (*p* < 0.0001 and *p* < 0.0001), respectively. As shown in Fig. 7c and d, the appearance of alveolar damage and fibrosis were also indicative of pulmonary fibrogenesis induced by PQ treatment. Furthermore, the levels of S100A8 and S100A9 expression in PQ-treated rat serum were also examined by ELISA analysis (Fig. S7†). A significant difference between the control group and PQ poisoning group was found in the serum concentrations of S100A8 (59.6 ± 11.7 pg mL^−1^ in the control group and 321.7 ± 37.2 pg mL^−1^ in the PQ poisoning group, *p* = 0.0025) and S100A9 (1.88 ± 0.37 ng mL^−1^ in the control group and 15.0 ± 1.31 ng mL^−1^ in the PQ poisoning group, *p* = 0.0006). These findings indicate that the upregulation of S100A8 and S100A9 in the lung tissue of PQ-treated rat was highly associated with lung damage and pulmonary fibrogenesis.

## 4. Discussion

Serum is the most important part for non-invasive biomarkers, which are ideal for disease prognosis, staging and monitoring. The identification of proteins that are significantly expressed between a population of cases and suitable control is an important step for the discovery of the biomarkers in serum. iTRAQ-coupled LC-MS/MS is a popular technique to undertake a high-throughput proteomics study to identify and quantify proteins differentially in abundance among disease sample and healthy control. To further improve the sensitivity and depth of differentially expressed proteins in serum/plasma by the iTRAQ-LC-MS/MS, Carr *et al.* applied the new workflow to a time series of plasma samples.^18^ In a very large-scape study using immunoaffinity depletion of the fourteen most abundant plasma proteins, 4-plex iTRAQ labelling of peptides obtained from the digestion of protein with trypsin and lysine-C, and LC-MS/MS analysis of 30 peptide fractions with 150 min gradient (210 min inject-to-inject), a core set of 3400 proteins was quantified in common across all 16 patients samples. Compared with a previously published label-free approach, the new workflow quantified almost five fold more proteins/sample and provided a six- to nine-fold increase in sample analysis throughput. Therefore, the iTRAQ coupled LC-MS/MS approach is the quantitative and efficient approach for biomarker discovery in plasma and serum.

Here, PQ poisoning remains a major public health concern in many countries. Due to the high rate of mortality associated with PQ poisoning, prompt diagnosis of PQ exposure is crucial for patient survival. Thus, in order to identify potential diagnostic biomarkers of PQ poisoning, serum samples from patients with PQ poisoning and healthy controls were labeled with iTRAQ reagents and analyzed by LC-MS/MS. A total of 81 differentially expressed proteins were identified in the sera of patients with PQ poisoning. Among these proteins, 30 and 51 proteins were significantly up- and down-regulated, respectively, in sera from patients with PQ poisoning. To the best of our knowledge, in our differentially expressed protein profiles, most proteins have not been reported in PQ poisoning previously. These proteins are mainly involved in important biologic processes that are closely associated with protein binding, enzyme regulator activity, inhibitor activity, and receptor activity, among others. According to the IPA analysis data, the most interesting finding of this study is that two of the up-regulated proteins and two of the down-regulated proteins identified through our comparative proteomics and validation approach, including S100A8, S100A9, TfR1, and SAP. We believe these candidate proteins may have potential as biomarkers for the diagnosis and therapy of PQ poisoning. Validation of these biomarkers and assessment of their changes in expression will provide useful information for the diagnosis and therapy of PQ poisoning.

It is important to note that the up-regulated of S100A8 and S100A9 are observed in PQ poisoning patients compared with normal controls. The up-regulation of S100A8 and S100A9 could be a potential diagnostic biomarker for PQ poisoning. Belonging to the S100 family of proteins, S100A8 and S100A9 are small calcium-binding proteins abundantly expressed by neutrophils, monocytes, and activated endothelial and epithelial cells.^19,20^ Generally, calcium-binding proteins act as intracellular calcium receptor molecules and couple changes in intracellular calcium levels to alterations in cell function. In addition to exhibiting a very complex mechanism of tissue/cell type-specific gene regulation, the S100 family of proteins appears to be a target of altered gene regulation in disease states and by pharmacological agents. Recent data has suggested that both proteins are among the most up-regulated genes in a number of acute and chronic inflammatory diseases, including cystic fibrosis, chronic inflammatory bowel disorder, Kawasaki disease and systemic lupus erythematosus, allergic dermatitis, and infection.^21–24^ Abnormal expression of S100A8 and S100A9 has been reported in various cancers, such as breast, lung, gastric, and colorectal cancer.^25–27^ However, the clinical importance of S100A8 and S100A9 expression in PQ poisoning has not previously been investigated. Our ROC curve analysis showed that measurement of the serum levels of S100A8 and S100A9 could provide a discriminatory diagnostic test for the presence of PQ poisoning.

Recruitment and stimulation of immune cells was active by S100A8 and S100A9 to produce pro-inflammatory cytokines, including TNF-α, IL-6, and IL-8. S100A8 and S100A9 act on TLR4 on immune cells, leading to the production of TNF-α and other inflammatory mediators such as cytokines during the inflammatory process.^28^ Alterations in pathways that perpetuate inflammation were also observed in our IPA results. Although there are various initiating mechanisms, pulmonary fibrosis is a frequent response to acute or chronic oxidative stress to the lungs. It is well-established that PQ poisoning can cause acute oxidative stress and severe lung injury during the exposure, finally leading to irreversible pulmonary fibrosis.^29^ To the best of our knowledge, this is the first investigation that focused on relevance of S100A8, S100A9 to PQ-induced pulmonary fibrosis in humans and rat. Pulmonary fibrosis in the lung tissue of PQ-treated rat was associated with interstitial inflammation and accumulated collagen synthesis, as well as a significant increase in the expression of S100A8 and S100A9. Thus, S100A8 and S100A9 may play roles in fibroblast growth-stimulating activity.

Some cytokines, including TNF-α, IL-6, and IL-8 were reported as clinically relevant therapeutic targets in PQ-induced inflammation.^30,31^ It has reported that the reduced levels of these pro-inflammatory cytokines found to decrease the severity of PQ-induced lung injury.^32^ S100A8 and S100A9, which are members of the alarmin family that are released into the extracellular space upon infection or tissue injury, were reported to lead to the production of TNF-α and other cytokines. Blockade of S100A8 and S100A9 suppressed proinflammatory activities, which suggested these alarmins may promote activation of innate immune cells and recruitment of antigen-presenting cells engaged in host defense and tissue repair. Our research suggests the upstream S100A8 and S100A9 signaling pathway are potential therapeutic targets for PQ-induced pathophysiological processes.

TfR1 is a membrane-associated glycoprotein receptor, also known as a key regulator of cellular iron homeostasis.^33^ TfR1 functions as a key port of iron acquisition by internalizing iron bound transferrin. TfR1 levels are tightly regulated by intracellular iron status. Thus, the expression level of TfR1 is sensitive to cellular iron status, being up-regulated in response to iron deficiency and down-regulated when iron levels are high.^34^ The expression of TfR1 has been found to increase in iron deficiency anaemia and in disorders that are associated with expanded erythropoiesis or ineffective erythropoiesis.^35,36^ The level of TfR in the plasma has been shown to be closely related to the number of red blood cell precursors in the bone marrow, and to provide a non-invasive reliable quantitative measure of the rate of total erythropoiesis in the absence of iron deficiency. Besides its essential role in iron uptake, TfR1 is intimately implicated in cell proliferation. Enhanced TfR1 expression is observed in proliferating cells and cancers. Previous studies have suggested that TfR1 could be a marker of malignant transformation in the pancreas.^37^ Herein, we report, for the first time, an apparent decrease in the expression of TfR1 in PQ poisoning. In this study, TfR1 discriminated PQ poisoning patients from normal controls with 71.4% sensitivity and 100% specificity. Owing to the redox-cycling activity of PQ, the herbicide is believed to exert its cytotoxic effects *via* oxidative stress.^38^ The mechanism that has been proposed for PQ redox cycling involves the enzymatic reduction of PQ to a cationic radical (PQ^+^*), which can subsequently reduce molecular oxygen (O_2_) to form the superoxide radical (O_2_^−^*) while also regenerating the parent compound (Fig. S8†). Previous studies have indicated that mitochondria may be involved in reactive oxygen species (ROS) generation by PQ. The production of ROS can overwhelm the anti-oxidant capacity of the cell, resulting in a state of oxidative stress and cytotoxicity. Under certain conditions, iron can catalyze the Fenton reaction that transforms H_2_O_2_ or lipid peroxides into the highly toxic hydroxyl radical OH* or lipid radicals, such as LO* and LOO*, thereby exacerbating cellular oxidative stress.^39^ Therefore, ROS can further effect cellular iron metabolism and aggravate the vicious circle of Fe/ROS-induced cellular damage. Treatment with iron chelating drugs has been shown to mitigate the cellular damage caused by free radicals.^40,41^ Thus, iron chelation maybe a promising therapeutic strategy to protect cells against damages induced by the redox-cycler PQ. As TfR1 has not yet been reported in PQ poisoning, its potential for improving the diagnosis, management, and treatment of PQ poisoning should be further examined.

Serum amyloid P (SAP) is a pentameric protein belonging to the pentraxin family of evolutionarily conserved proteins that includes C-reactive protein (CRP) and pentraxin-3 (PTX3).^42^ SAP and CRP are pattern recognition molecules secreted by the liver, which interact with pathogens and cell debris to promote their removal by macrophages and neutrophils.^43^ SAP regulates many aspects of neutrophil biology to exert its anti-inflammatory effects, and sets a threshold for neutrophil recruitment and activation.^44^ Additionally, SAP and CRP interact with components of the complement pathway to regulate complement activation and phagocytosis. Furthermore, SAP is a key ligand that acts on monocytes, neutrophils, and macrophages to modify their activation; thereby altering their differentiation in order to modulate the immune response and regulate inflammation and fibrosis.^45,46^ Thus, injections of human or mouse SAP can inhibit inflammation and fibrosis in animal models of pulmonary fibrosis, cardiac fibrosis, dermal wound healing, radiation-induced oral mucositis, allergic airway disease, experimental autoimmune encephalomyelitis, and kidney injury.^47–49^ However, the clinical importance of SAP expression in PQ poisoning has rarely been addressed. In our study, the expression of SAP was found to decrease in the serum of patients with PQ poisoning and this down-regulation may be associated with liver toxicity induced by PQ. However, the potential contribution of SAP to PQ intoxication remains to be elucidated.

## 5. General conclusion

In summary, this study demonstrated that S100A8 and S100A9 are overexpressed in the sera of PQ poisoning human patients and PQ-treated rat. In the rat model, lung tissue expression levels of S100A8 and S100A9 are also positively correlated with PQ poisoning. These two S100 proteins show great advantages over traditional clinical or laboratory markers for specific indications due to their local expression and release in direct response to tissue damage. They may be trigger alarmins involved in markers of early and reliable diagnosis of PQ poisoning. Thus, S100A8 and S100A9 are associated with lung damage and pulmonary fibrosis by PQ poisoning, and then the mechanism and the potential roles of S100A8 and S100A9 in PQ poisoning require urgent clarification. Furthermore, the other two interesting proteins, the down-regulation of serum TfR1 and SAP concentration is significantly highlighted by our results in PQ poisoning patients. In addition, more speculatively, the TfR1 and SAP may be the novel therapeutic target, as expression levels and correlation to PQ poisoning are unusually strong, suggesting causal links. Further investigation will shed light on the validity of this notion.

## Author contributions

P. Y. and M. S. conceived the studies. P. Y. and S. C. designed the studies. L. W. and Y. W. conducted experiments. L. W. and L. Z. analyzed the results. L. W., Y. W., L. L., L. Z., Y. S., P. X., H. S. wrote or helped to draft the manuscript. All authors have reviewed the manuscript.

## Conflicts of interest

The authors declare no competing financial interests.

## Supplementary Material

RA-008-C7RA12956D-s001
