# Correction: Identification of potential serum biomarkers of acute paraquat poisoning in humans using an iTRAQ quantitative proteomic

**DOI:** 10.1039/c9ra90006c

**Published:** 2019-01-28

**Authors:** Liming Wei, Yi Wang, Ling Lin, Lei Zhang, Yan Shi, Ping Xiang, Shujun Cao, Min Shen, Pengyuan Yang

**Affiliations:** Shanghai Key Laboratory of Forensic Medicine, Shanghai Forensic Service Platform, Institute of Forensic Science, Ministry of Justice Shanghai China shenm@ssfjd.cn; Institutes of Biomedical Sciences, Department of Chemistry, Fudan University Shanghai China pyyang@fudan.edu.cn; Shanghai Songjiang District Central Hospital Shanghai China shujun-cao@163.com

## Abstract

Correction for ‘Identification of potential serum biomarkers of acute paraquat poisoning in humans using an iTRAQ quantitative proteomic’ by Liming Wei *et al.*, *RSC Adv.*, 2018, 8, 10598–10609.

We regret that two of the corresponding authors, Shujun Cao and Min Shen, were not identified explicitly in the original manuscript. The corrected list of corresponding authors for this paper is as shown above.

The Royal Society of Chemistry apologises for these errors and any consequent inconvenience to authors and readers.

## Supplementary Material

