# Supplementary material for: Identification of potential serum biomarkers of acute paraquat poisoning in humans using an iTRAQ quantitative proteomic
Source: RSC Adv. 2018 Mar 16;8(19):10598–609. doi: 10.1039/c7ra12956d (PMC9078879; doi:10.1039/c7ra12956d)
Supplement: RA-008-C7RA12956D-s001 [file RA-008-C7RA12956D-s001.pdf]

Electronic Supplementary Material (ESI) for RSC Advances.
This journal is © The Royal Society of Chemistry 2018

Supplementary information

Identification potential serum biomarkers of acute paraquat poisoning in humans using iTRAQ quantitative proteomic

Liming Wei[2,3#], Yi Wang[2#], Ling Lin[2], Lei Zhang[2], Yan Shi[1], Ping Xiang[1], Shujun Cao[3], Min Shen[1], Pengyuan Yang[2]

[1] Shanghai Key Laboratory of Forensic Medicine, Shanghai Forensic Service Platform, Institute of Forensic Science, Ministry of Justice, Shanghai, China

[2] Institutes of Biomedical Sciences & Department of Chemistry, Fudan University, Shanghai, China

[3] Shanghai Songjiang District Central Hospital, Shanghai, China.

[#]These authors contributed equally to this work. Correspondence and requests for materials should be addressed to P.Y. (email: pyyang@fudan.edu.cn), M.S. (email: shenm@ssfjd.cn) or S.C. (email: shujun-cao@163.com)

Table S1. Detailed clinical information of each cohort

	PQ poisoning patients	Healthy controls
Sample size	21	21
Sex (M/F)	8/13	10/11
Age (year)	35.5±12.3	32.5±8.5
PQ plasma concentration (ug/mL)	1.05±3.50	No signal

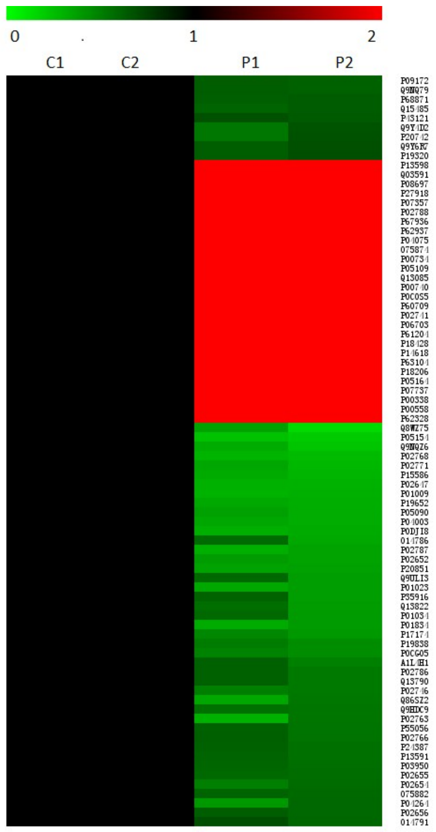

Fig. S1. Hierarchical clustering for differentially expressed proteins in PQ poisoning sample. Hierarchical clustering was performed with correlations on the proteins used to generate the heat map using the MultiExperiment Viewer. The proteins in clusters exhibit progressive up- or down-regulated patternsin PQ poisoning and were grouped into two representative clusters, Clusters 1-4. C1 and C2 represent two technical replicates of the iTRAQ-LC-MS/MS procedure from the healthy control cohort, while P1 and P2 represent two technical replicates from the cohort of PQ poisoning patients.

Table S2. A list of identified proteins (413 proteins) in combination of two technical replicates from PQ poisoning patients and healthy controls

Accession	Description	Coverage	# Unique Peptides	# Peptides	# PSMs	114/113
P62258	14-3-3 protein epsilon	10.59	1	2	3	3.703
P61981	14-3-3 protein gamma	10.93	1	2	3	1.064
P61981	14-3-3 protein gamma	8.91	1	2	3	0.956
P63104	14-3-3 protein zeta/delta	21.63	3	4	6	2.446
P63104	14-3-3 protein zeta/delta	21.63	3	4	6	2.329
Q9NRZ5	1-acyl-sn-glycerol-3-phosphate acyltransferase delta	1.85	1	1	1	1.148
P62191	26S protease regulatory subunit 4	2.05	1	1	1	0.993
Q3SYC2	2-acylglycerol	2.69	1	1	1	1.223
P08253	72 kDa type IV collagenase	1.82	1	1	1	0.536
P11021	78 kDa glucose-regulated protein	7.03	3	3	3	0.711
P11021	78 kDa glucose-regulated protein	4.43	2	2	2	0.759
Q76LX8	A disintegrin and metalloproteinase with thrombospondin motifs 13	2.31	3	3	4	0.892
Q76LX8	A disintegrin and metalloproteinase with thrombospondin motifs 13	3.01	4	4	6	1.218
Q13085	Acetyl-CoA carboxylase 1	0.30	1	1	1	2.046
Q13085	Acetyl-CoA carboxylase 1	0.30	1	1	3	1.843
Q99798	Aconitate hydratase, mitochondrial	0.77	1	1	1	1.001
P60709	Actin, cytoplasmic 1	26.67	9	9	20	2.143
P60709	Actin, cytoplasmic 1	26.67	9	9	18	**2.058**
Q9NZK5	Adenosine deaminase CECR1	1.57	1	1	1	0.927
Q01518	Adenylyl cyclase-associated protein 1	2.74	1	1	1	0.936
Q01518	Adenylyl cyclase-associated protein 1	2.74	1	1	1	1.035
Q9HDC9	Adipocyte plasma membrane-associated protein	1.68	1	1	1	0.534
Q9HDC9	Adipocyte plasma membrane-associated protein	1.68	1	1	1	0.549
Q15848	Adiponectin	6.15	1	1	1	0.840
Q15848	Adiponectin	6.15	1	1	1	0.857
P61204	ADP-ribosylation factor 3	3.87	1	1	1	2.182
P61204	ADP-ribosylation factor 3	3.87	1	1	1	1.871
P43652	Afamin	34.06	21	21	55	0.960
P43652	Afamin	39.90	24	24	60	1.108
Q13023	A-kinase anchor protein 6	0.30	1	1	1	1.029
P02763	Alpha-1-acid glycoprotein 1	15.92	2	3	7	0.536
P02763	Alpha-1-acid glycoprotein 1	34.83	4	6	10	0.302
P19652	Alpha-1-acid glycoprotein 2	16.42	2	3	8	0.314
P19652	Alpha-1-acid glycoprotein 2	35.32	4	6	10	0.334
P01011	Alpha-1-antichymotrypsin	34.28	14	14	74	1.043
P01011	Alpha-1-antichymotrypsin	35.93	15	15	87	1.134
P01009	Alpha-1-antitrypsin	53.83	20	20	98	**0.307**
P01009	Alpha-1-antitrypsin	53.83	22	22	102	**0.297**
P04217	Alpha-1B-glycoprotein	33.94	14	14	81	1.066
P04217	Alpha-1B-glycoprotein	33.94	14	14	101	1.077
P08697	Alpha-2-antiplasmin	24.64	11	11	23	1.614
P08697	Alpha-2-antiplasmin	27.29	10	10	27	1.610
P02765	Alpha-2-HS-glycoprotein	45.50	13	13	81	1.413

P02765	Alpha-2-HS-glycoprotein	45.50	13	13	73	1.409
P01023	Alpha-2-macroglobulin	25.31	25	29	47	**0.378**
P01023	Alpha-2-macroglobulin	22.32	24	28	45	**0.338**
P12814	Alpha-actinin-1	2.58	2	2	3	1.418
P12814	Alpha-actinin-1	5.72	3	3	3	2.886
P06733	Alpha-enolase	1.84	1	1	1	1.124
P06733	Alpha-enolase	1.84	1	1	1	1.575
P02771	Alpha-fetoprotein	1.15	1	1	1	0.277
P02771	Alpha-fetoprotein	1.15	1	1	1	0.342
Q16706	Alpha-mannosidase 2	0.61	1	1	1	1.533
Q16706	Alpha-mannosidase 2	0.61	1	1	1	1.302
Q03154	Aminoacylase-1	1.96	1	1	1	0.744
P15144	Aminopeptidase N	3.31	3	3	3	0.738
P15144	Aminopeptidase N	0.93	1	1	1	0.792
P05067	Amyloid beta A4 protein	1.56	1	1	1	0.741
P03950	Angiogenin	13.61	1	1	1	0.565
P03950	Angiogenin	13.61	1	1	1	0.598
P12821	Angiotensin-converting enzyme	0.54	1	1	1	0.749
P01019	Angiotensinogen	19.79	9	9	33	1.255
P01019	Angiotensinogen	21.65	9	9	37	1.196
Q8IWZ3	Ankyrin repeat and KH domain-containing protein 1	0.39	1	1	1	1.062
P12429	Annexin A3	2.79	1	1	1	2.636
P01008	Antithrombin-III	44.83	23	23	82	1.393
P01008	Antithrombin-III	40.52	23	23	79	1.347
P02647	Apolipoprotein A-I	66.29	18	18	111	**0.299**
P02647	Apolipoprotein A-I	60.67	17	17	110	**0.305**
P02652	Apolipoprotein A-II	62.00	5	5	19	0.354
P02652	Apolipoprotein A-II	64.00	6	6	13	0.385
P06727	Apolipoprotein A-IV	52.53	21	21	63	1.099
P06727	Apolipoprotein A-IV	60.10	27	27	70	1.201
P04114	Apolipoprotein B-100	41.62	167	167	460	0.940
P04114	Apolipoprotein B-100	44.12	173	173	498	0.898
P02654	Apolipoprotein C-I	30.12	3	3	4	0.573
P02654	Apolipoprotein C-I	19.28	2	2	7	0.501
P02655	Apolipoprotein C-II	49.50	4	4	6	0.571
P02655	Apolipoprotein C-II	28.71	3	3	5	0.584
P02656	Apolipoprotein C-III	27.27	2	2	10	0.592
P02656	Apolipoprotein C-III	34.34	3	3	7	0.629
P55056	Apolipoprotein C-IV	12.60	2	2	2	0.544
P55056	Apolipoprotein C-IV	12.60	2	2	2	0.615
P05090	Apolipoprotein D	12.70	2	2	2	0.318
P05090	Apolipoprotein D	12.70	2	2	2	0.366
P02649	Apolipoprotein E	58.99	16	16	33	0.922
P02649	Apolipoprotein E	63.41	19	19	39	0.933
Q13790	Apolipoprotein F	8.28	2	2	3	0.521
Q13790	Apolipoprotein F	8.28	2	2	4	0.619
O14791	Apolipoprotein L1	22.11	6	6	7	0.599
O14791	Apolipoprotein L1	16.58	5	5	5	0.687
O95445	Apolipoprotein M	15.96	3	3	5	0.873
O95445	Apolipoprotein M	12.23	2	2	3	0.909
P08519	Apolipoprotein(a)	7.50	2	3	6	2.631
P08519	Apolipoprotein(a)	7.15	1	2	5	1.727
P17174	Aspartate aminotransferase, cytoplasmic	2.18	1	1	1	0.393

P17174	Aspartate aminotransferase, cytoplasmic	2.18	1	1	1	0.466
O75882	Attractin	8.33	9	9	12	0.584
O75882	Attractin	5.95	8	8	13	0.603
O14645	Axonemal dynein light intermediate polypeptide 1	2.33	1	1	1	0.548
P20160	Azurocidin	3.98	1	1	1	0.823
P98160	Basement membrane-specific heparan sulfate proteoglycan core protein	0.48	2	2	2	0.440
P98160	Basement membrane-specific heparan sulfate proteoglycan core protein	0.43	2	2	2	0.903
O43286	Beta-1,4-galactosyltransferase 5	2.06	1	1	1	
P02749	Beta-2-glycoprotein 1	45.80	12	12	27	1.509
P02749	Beta-2-glycoprotein 1	36.52	12	12	33	1.406
P61769	Beta-2-microglobulin	16.81	2	2	2	0.640
P61769	Beta-2-microglobulin	35.29	3	3	3	0.696
Q96KN2	Beta-Ala-His dipeptidase	10.26	5	5	6	0.844
Q96KN2	Beta-Ala-His dipeptidase	15.58	7	7	10	1.012
P43251	Biotinidase	13.26	6	6	11	1.036
P43251	Biotinidase	10.68	5	5	11	1.292
P13727	Bone marrow proteoglycan	8.11	1	1	1	1.323
P13727	Bone marrow proteoglycan	8.11	1	1	1	0.583
P04003	C4b-binding protein alpha chain	11.89	7	7	13	0.320
P04003	C4b-binding protein alpha chain	14.57	9	9	12	0.342
P20851	C4b-binding protein beta chain	3.57	1	1	2	0.365
P20851	C4b-binding protein beta chain	3.57	1	1	1	0.355
P12830	Cadherin-1	1.59	1	1	1	0.656
P12830	Cadherin-1	1.59	1	1	1	0.683
P55290	Cadherin-13	3.65	2	2	3	0.889
P55290	Cadherin-13	6.87	4	4	5	0.660
P33151	Cadherin-5	1.66	1	1	1	1.275
P33151	Cadherin-5	2.68	2	2	2	1.130
Q9BYE9	Cadherin-related family member 2	0.99	1	1	1	0.663
P62158	Calmodulin	6.04	1	1	1	1.428
Q99439	Calponin-2	5.50	1	1	1	0.676
P27797	Calreticulin	2.88	1	1	1	1.168
P00915	Carbonic anhydrase 1	9.96	3	3	6	1.405
P00915	Carbonic anhydrase 1	9.96	3	3	6	1.190
P00918	Carbonic anhydrase 2	3.46	1	1	1	1.404
Q96IY4	Carboxypeptidase B2	19.15	9	9	17	1.140
Q96IY4	Carboxypeptidase B2	13.71	6	6	12	1.056
P15169	Carboxypeptidase N catalytic chain	18.56	8	8	11	0.929
P15169	Carboxypeptidase N catalytic chain	14.85	6	6	9	1.211
P22792	Carboxypeptidase N subunit 2	23.49	11	11	18	1.366
P22792	Carboxypeptidase N subunit 2	23.49	11	11	17	1.503
Q9Y646	Carboxypeptidase Q	1.91	1	1	1	0.724
Q9Y646	Carboxypeptidase Q	4.24	2	2	2	0.679
P31997	Carcinoembryonic antigen-related cell adhesion molecule 8	2.58	1	1	1	1.301
Q9NQ79	Cartilage acidic protein 1	5.14	2	2	2	0.615
Q9NQ79	Cartilage acidic protein 1	2.87	2	2	2	0.632
P49747	Cartilage oligomeric matrix protein	6.08	4	4	4	0.825
P49747	Cartilage oligomeric matrix protein	3.96	3	3	3	0.694
P04040	Catalase	10.06	4	4	4	1.089
P04040	Catalase	13.85	6	6	6	1.060

P08311	Cathepsin G	3.53	1	1	1	1.195
P08311	Cathepsin G	3.53	1	1	1	1.635
Q9UBR2	Cathepsin Z	3.96	1	1	1	1.005
P11717	Cation-independent mannose-6-phosphate receptor	0.28	1	1	1	0.974
Q6YHK3	CD109 antigen	0.69	1	1	1	1.152
Q6YHK3	CD109 antigen	1.66	2	2	2	1.164
P16070	CD44 antigen	2.83	2	2	4	1.417
P16070	CD44 antigen	2.83	2	2	4	1.499
O43866	CD5 antigen-like	2.02	1	1	2	1.052
O43866	CD5 antigen-like	2.02	1	1	1	1.372
P43121	Cell surface glycoprotein MUC18	5.88	3	3	3	0.640
P43121	Cell surface glycoprotein MUC18	1.39	1	1	1	0.675
P00450	Ceruloplasmin	38.12	35	35	124	1.202
P00450	Ceruloplasmin	39.62	35	35	122	1.361
Q96FZ7	Charged multivesicular body protein 6	2.99	1	1	1	2.269
O00299	Chloride intracellular channel protein 1	3.73	1	1	1	2.615
P06276	Cholinesterase	15.61	8	8	11	1.294
P06276	Cholinesterase	16.61	9	9	13	1.042
Q99895	Chymotrypsin-C	2.61	1	1	1	0.932
Q99895	Chymotrypsin-C	2.61	1	1	1	0.986
P10909	Clusterin	23.83	9	9	23	1.440
P10909	Clusterin	23.83	9	9	32	1.241
P00740	Coagulation factor IX	6.94	3	3	4	2.048
P00740	Coagulation factor IX	8.68	4	4	5	1.734
P12259	Coagulation factor V	4.99	12	12	15	0.931
P12259	Coagulation factor V	5.58	13	13	14	0.960
P08709	Coagulation factor VII	4.94	1	1	1	0.523
P08709	Coagulation factor VII	2.15	1	1	1	0.779
P00742	Coagulation factor X	16.19	6	6	8	1.009
P00742	Coagulation factor X	16.19	6	6	8	1.217
P03951	Coagulation factor XI	15.04	9	9	12	1.310
P03951	Coagulation factor XI	18.88	11	11	15	1.399
P00748	Coagulation factor XII	19.67	11	11	16	0.962
P00748	Coagulation factor XII	24.07	13	13	18	1.201
P00488	Coagulation factor XIII A chain	10.38	6	6	6	1.306
P00488	Coagulation factor XIII A chain	3.83	2	2	2	1.745
P05160	Coagulation factor XIII B chain	16.34	8	8	13	0.923
P05160	Coagulation factor XIII B chain	17.10	9	9	13	1.068
P23528	Cofilin-1	20.48	2	2	2	1.363
Q96EE4	Coiled-coil domain-containing protein 126	8.57	1	1	2	1.448
P12109	Collagen alpha-1(VI) chain	0.88	1	1	1	0.870
P39060	Collagen alpha-1(XVIII) chain	0.97	1	1	1	0.870
P39060	Collagen alpha-1(XVIII) chain	1.48	2	2	2	0.905
P12110	Collagen alpha-2(VI) chain	0.88	1	1	1	0.776
P12111	Collagen alpha-3(VI) chain	1.57	4	4	4	0.763
P12111	Collagen alpha-3(VI) chain	1.29	4	4	4	0.768
Q9Y6Z7	Collectin-10	3.61	1	1	1	1.071
Q9BWP8	Collectin-11	4.06	1	1	1	1.340
Q9BWP8	Collectin-11	3.69	1	1	1	1.070
P02745	Complement C1q subcomponent subunit A	13.47	2	2	3	0.986
P02745	Complement C1q subcomponent subunit A	4.08	1	1	1	1.038
P02746	Complement C1q subcomponent subunit B	8.70	2	2	5	0.525
P02746	Complement C1q subcomponent subunit B	8.70	3	3	6	0.500

P02747	Complement C1q subcomponent subunit C	25.31	5	5	9	0.771
P02747	Complement C1q subcomponent subunit C	25.31	5	5	10	0.891
P00736	Complement C1r subcomponent	28.94	13	15	24	1.419
P00736	Complement C1r subcomponent	28.79	16	17	28	1.309
Q9NZP8	Complement C1r subcomponent-like protein	10.68	4	6	8	1.296
Q9NZP8	Complement C1r subcomponent-like protein	8.62	4	5	7	0.803
P09871	Complement C1s subcomponent	22.53	11	12	20	0.833
P09871	Complement C1s subcomponent	23.98	11	12	21	0.908
P06681	Complement C2	23.40	19	19	37	1.011
P06681	Complement C2	21.01	16	16	42	0.841
P01024	Complement C3	19.18	28	28	47	1.388
P01024	Complement C3	18.88	29	29	49	1.410
P0C0L4	Complement C4-A	44.15	3	74	266	1.369
P0C0L4	Complement C4-A	44.67	3	72	276	1.366
P0C0L5	Complement C4-B	43.92	3	74	268	0.793
P0C0L5	Complement C4-B	45.13	4	73	278	0.767
P01031	Complement C5	24.11	42	42	84	0.965
P01031	Complement C5	26.13	44	44	85	0.912
Q9NPY3	Complement component C1q receptor	1.07	1	1	1	0.992
P13671	Complement component C6	21.73	19	19	41	1.430
P13671	Complement component C6	22.48	20	20	47	1.202
P10643	Complement component C7	22.89	18	18	38	0.992
P10643	Complement component C7	17.91	15	15	38	0.974
P07357	Complement component C8 alpha chain	19.52	9	9	14	1.717
P07357	Complement component C8 alpha chain	21.06	10	10	11	1.497
P07358	Complement component C8 beta chain	25.38	15	15	27	1.054
P07358	Complement component C8 beta chain	22.17	13	13	22	1.094
P07360	Complement component C8 gamma chain	58.42	9	9	16	1.275
P07360	Complement component C8 gamma chain	50.50	8	8	14	1.283
P02748	Complement component C9	28.98	16	16	36	1.383
P02748	Complement component C9	32.74	18	18	43	1.355
P00751	Complement factor B	41.88	33	33	95	1.296
P00751	Complement factor B	40.84	31	31	96	1.243
P00746	Complement factor D	15.42	3	3	4	0.893
P00746	Complement factor D	19.76	3	3	4	0.786
P08603	Complement factor H	41.75	41	44	108	1.165
P08603	Complement factor H	40.21	38	42	102	1.125
Q03591	Complement factor H-related protein 1	21.82	2	7	14	1.584
Q03591	Complement factor H-related protein 1	25.15	2	8	15	1.460
P36980	Complement factor H-related protein 2	23.33	3	6	10	1.799
P36980	Complement factor H-related protein 2	23.33	3	6	10	1.260
P05156	Complement factor I	20.24	10	10	18	1.294
P05156	Complement factor I	18.18	9	9	17	1.216
Q12860	Contactin-1	0.98	1	1	1	0.740
P08185	Corticosteroid-binding globulin	22.22	7	7	18	0.718
P08185	Corticosteroid-binding globulin	14.32	5	5	17	0.837
P24387	Corticotropin-releasing factor-binding protein	3.11	1	1	1	0.554
P24387	Corticotropin-releasing factor-binding protein	3.11	1	1	1	0.616
Q9P1F3	Costars family protein ABRACL	16.05	1	1	1	1.697
P02741	C-reactive protein	20.98	5	5	13	2.167
P02741	C-reactive protein	24.11	6	6	14	2.250
Q9UBG0	C-type mannose receptor 2	0.88	1	1	1	1.030
P01034	Cystatin-C	7.53	1	1	1	0.389
P01034	Cystatin-C	7.53	1	1	1	0.588

P54108	Cysteine-rich secretory protein 3	6.53	2	2	3	1.357
P54108	Cysteine-rich secretory protein 3	3.27	1	1	2	1.090
Q07065	Cytoskeleton-associated protein 4	1.16	1	1	1	0.528
P81605	Dermcidin	10.00	1	1	1	0.843
P81605	Dermcidin	10.00	1	1	1	0.736
Q14126	Desmoglein-2	0.89	1	1	1	1.111
Q14126	Desmoglein-2	0.89	1	1	1	1.075
Q01459	Di-N-acetylchitobiase	5.97	2	2	2	0.695
Q01459	Di-N-acetylchitobiase	5.97	2	2	2	0.835
Q9H4A9	Dipeptidase 2	2.06	1	1	1	1.000
Q9H4A9	Dipeptidase 2	2.06	1	1	1	0.731
P27487	Dipeptidyl peptidase 4	6.79	4	4	4	1.171
P27487	Dipeptidyl peptidase 4	4.05	3	3	3	1.460
P09172	Dopamine beta-hydroxylase	5.35	3	3	7	0.612
P09172	Dopamine beta-hydroxylase	6.16	4	4	9	0.629
Q96L15	Ecto-ADP-ribosyltransferase 5	2.06	1	1	1	
Q96L15	Ecto-ADP-ribosyltransferase 5	2.06	1	1	1	
Q13822	Ectonucleotide pyrophosphatase/phosphodiesterase family member 2	2.67	1	1	1	0.386
Q13822	Ectonucleotide pyrophosphatase/phosphodiesterase family member 2	6.49	4	4	4	0.565
Q12805	EGF-containing fibulin-like extracellular matrix protein 1	4.67	1	1	1	1.519
Q7Z2Z2	Elongation factor Tu GTP-binding domain-containing protein 1	0.63	1	1	1	0.309
Q9NZ08	Endoplasmic reticulum aminopeptidase 1	1.38	1	1	1	1.206
Q9NZ08	Endoplasmic reticulum aminopeptidase 1	1.49	1	1	1	0.655
Q6P179	Endoplasmic reticulum aminopeptidase 2	1.35	1	1	1	0.927
P14625	Endoplasmin	1.37	1	1	1	0.303
Q9UNN8	Endothelial protein C receptor	3.78	1	1	1	0.630
Q9UNN8	Endothelial protein C receptor	3.78	1	1	1	0.711
Q9UBQ6	Exostosin-like 2	2.12	1	1	1	0.938
Q9UBQ6	Exostosin-like 2	2.12	1	1	1	1.018
Q16610	Extracellular matrix protein 1	17.78	8	8	10	1.131
Q16610	Extracellular matrix protein 1	13.33	7	7	9	1.552
Q8IXL6	Extracellular serine/threonine protein kinase FAM20C	1.20	1	1	1	0.903
P08294	Extracellular superoxide dismutase [Cu-Zn]	8.75	2	2	2	0.998
P08294	Extracellular superoxide dismutase [Cu-Zn]	3.33	1	1	1	2.486
Q96AE4	Far upstream element-binding protein 1	1.09	1	1	1	2.456
Q86UX7	Fermitin family homolog 3	6.00	3	3	4	3.104
Q86UX7	Fermitin family homolog 3	8.25	5	5	6	2.186
Q9UGM5	Fetuin-B	18.32	6	6	9	1.219
Q9UGM5	Fetuin-B	21.73	6	6	7	1.126
P02671	Fibrinogen alpha chain	12.47	9	9	23	0.876
P02671	Fibrinogen alpha chain	13.39	10	10	16	1.155
P02675	Fibrinogen beta chain	6.92	4	4	4	0.880
P02675	Fibrinogen beta chain	9.16	6	6	7	1.174
P02679	Fibrinogen gamma chain	1.99	1	1	1	0.803
P02679	Fibrinogen gamma chain	1.99	1	1	1	0.947
P02751	Fibronectin	8.21	15	15	21	1.040
P02751	Fibronectin	9.51	17	17	23	1.320

P23142	Fibulin-1	6.97	4	4	6	1.233
P23142	Fibulin-1	8.39	5	5	5	1.173
Q15485	Ficolin-2	5.11	2	2	2	0.639
Q15485	Ficolin-2	2.88	1	1	2	0.603
O75636	Ficolin-3	34.11	10	10	24	0.920
O75636	Ficolin-3	28.76	8	8	20	0.922
P21333	Filamin-A	0.53	1	1	1	1.079
P30043	Flavin reductase (NADPH)	7.28	1	1	2	1.854
P30043	Flavin reductase (NADPH)	7.28	1	1	2	0.820
B1AJZ9	Forkhead-associated domain-containing protein 1	1.49	1	1	1	0.608
P04075	Fructose-bisphosphate aldolase A	2.20	1	1	1	1.970
P04075	Fructose-bisphosphate aldolase A	6.59	2	3	3	1.815
P05062	Fructose-bisphosphate aldolase B	7.14	2	2	2	1.305
P05062	Fructose-bisphosphate aldolase B	9.07	2	3	3	1.307
P16930	Fumarylacetoacetase	3.10	1	1	1	1.608
P16930	Fumarylacetoacetase	10.26	4	4	4	0.661
Q08380	Galectin-3-binding protein	10.26	5	5	5	1.074
Q08380	Galectin-3-binding protein	10.26	5	5	7	0.870
Q92820	Gamma-glutamyl hydrolase	9.12	2	2	3	1.213
Q92820	Gamma-glutamyl hydrolase	4.40	1	1	1	0.775
P06396	Gelsolin	32.86	23	23	43	1.017
P06396	Gelsolin	37.85	24	24	49	1.008
P06744	Glucose-6-phosphate isomerase	1.43	1	1	1	0.530
Q07075	Glutamyl aminopeptidase	1.36	1	1	1	0.721
P22352	Glutathione peroxidase 3	15.93	3	3	5	0.763
P22352	Glutathione peroxidase 3	15.93	3	3	6	0.845
P48637	Glutathione synthetase	3.59	1	1	1	0.310
Q9H583	HEAT repeat-containing protein 1	0.37	1	1	1	0.453
P08107	Heat shock 70 kDa protein 1A/1B	3.90	1	2	2	1.575
P11142	Heat shock cognate 71 kDa protein	6.97	2	3	3	2.489
P69905	Hemoglobin subunit alpha	8.45	1	1	1	0.741
P68871	Hemoglobin subunit beta	27.89	4	4	5	0.639
P68871	Hemoglobin subunit beta	27.89	4	4	7	0.625
P02790	Hemopexin	52.81	21	21	118	0.858
P02790	Hemopexin	62.12	23	23	134	0.926
P05546	Heparin cofactor 2	18.24	11	11	28	1.143
P05546	Heparin cofactor 2	26.65	15	15	42	1.178
Q04756	Hepatocyte growth factor activator	9.01	5	5	7	1.353
Q04756	Hepatocyte growth factor activator	10.69	6	6	8	1.436
P08581	Hepatocyte growth factor receptor	0.65	1	1	1	0.716
P26927	Hepatocyte growth factor-like protein	5.63	4	4	5	1.333
P26927	Hepatocyte growth factor-like protein	8.72	6	6	7	1.095
O42043	HERV-K_1q23.3 provirus ancestral Env polyprotein	1.61	1	1	1	0.463
P04196	Histidine-rich glycoprotein	25.52	15	15	37	0.694
P04196	Histidine-rich glycoprotein	22.48	14	14	41	0.672
P0C0S5	Histone H2A.Z	5.47	1	1	1	2.062
P0C0S5	Histone H2A.Z	5.47	1	1	1	2.064
P62805	Histone H4	9.71	1	1	1	0.936
Q92839	Hyaluronan synthase 1	1.38	1	1	1	0.614
Q14520	Hyaluronan-binding protein 2	9.11	6	6	7	0.711
Q14520	Hyaluronan-binding protein 2	9.11	6	6	7	0.843
Q9Y4L1	Hypoxia up-regulated protein 1	0.80	1	1	1	1.253

P01859	Ig gamma-2 chain C region	3.07	1	1	1	0.593
P01861	Ig gamma-4 chain C region	7.95	2	2	2	0.530
P01834	Ig kappa chain C region	15.09	1	1	1	0.390
P01834	Ig kappa chain C region	15.09	1	1	1	0.323
P01603	Ig kappa chain V-I region Ka	14.81	1	1	2	0.324
P0CG05	Ig lambda-2 chain C regions	9.43	1	1	1	0.444
P0CG05	Ig lambda-2 chain C regions	9.43	1	1	1	0.486
Q9Y6R7	IgGFc-binding protein	1.22	3	3	3	0.683
Q9Y6R7	IgGFc-binding protein	1.46	4	4	5	0.632
P01344	Insulin-like growth factor II	8.89	1	1	1	0.795
P18065	Insulin-like growth factor-binding protein 2	2.77	1	1	1	1.285
P17936	Insulin-like growth factor-binding protein 3	19.93	5	5	7	1.630
P17936	Insulin-like growth factor-binding protein 3	18.90	5	5	8	1.355
P24592	Insulin-like growth factor-binding protein 6	2.50	1	1	1	
P35858	Insulin-like growth factor-binding protein complex acid labile subunit	37.85	19	19	49	1.070
P35858	Insulin-like growth factor-binding protein complex acid labile subunit	44.46	22	22	53	1.190
P19827	Inter-alpha-trypsin inhibitor heavy chain H1	23.16	17	17	70	1.061
P19827	Inter-alpha-trypsin inhibitor heavy chain H1	24.48	18	18	84	0.990
P19823	Inter-alpha-trypsin inhibitor heavy chain H2	27.06	27	27	127	1.152
P19823	Inter-alpha-trypsin inhibitor heavy chain H2	29.70	28	28	122	1.228
Q06033	Inter-alpha-trypsin inhibitor heavy chain H3	10.34	8	8	16	0.911
Q06033	Inter-alpha-trypsin inhibitor heavy chain H3	11.57	9	9	18	1.179
Q14624	Inter-alpha-trypsin inhibitor heavy chain H4	39.03	30	30	98	1.205
Q14624	Inter-alpha-trypsin inhibitor heavy chain H4	43.98	31	31	91	1.193
P05362	Intercellular adhesion molecule 1	4.51	2	2	2	0.852
P13598	Intercellular adhesion molecule 2	2.55	1	1	1	1.570
P13598	Intercellular adhesion molecule 2	2.55	1	1	1	1.637
P32942	Intercellular adhesion molecule 3	1.65	1	1	1	1.173
P32942	Intercellular adhesion molecule 3	1.65	1	1	1	1.363
Q9NPH3	Interleukin-1 receptor accessory protein	6.32	4	4	6	0.745
Q9NPH3	Interleukin-1 receptor accessory protein	8.07	5	5	5	0.698
P40189	Interleukin-6 receptor subunit beta	0.87	1	1	1	0.848
O75874	Isocitrate dehydrogenase [NADP] cytoplasmic	2.17	1	1	1	1.991
O75874	Isocitrate dehydrogenase [NADP] cytoplasmic	2.17	1	1	1	2.335
P29622	Kallistatin	32.79	12	12	20	1.030
P29622	Kallistatin	39.58	15	15	26	1.079
P13645	Keratin, type I cytoskeletal 10	5.82	2	2	2	0.437
P04264	Keratin, type II cytoskeletal 1	1.40	1	1	1	0.588
P04264	Keratin, type II cytoskeletal 1	2.33	1	1	1	0.398
P01042	Kininogen-1	28.42	20	20	76	1.028
P01042	Kininogen-1	31.06	20	20	77	1.059
P02788	Lactotransferrin	3.24	2	2	4	1.737
P02788	Lactotransferrin	9.30	6	6	7	1.746
P02750	Leucine-rich alpha-2-glycoprotein	44.09	11	11	28	1.237
P02750	Leucine-rich alpha-2-glycoprotein	48.13	13	13	37	1.254
P30740	Leukocyte elastase inhibitor	3.17	1	1	1	1.046
P30740	Leukocyte elastase inhibitor	3.17	1	1	1	0.674
P18428	Lipopolysaccharide-binding protein	17.26	5	5	9	2.229
P18428	Lipopolysaccharide-binding protein	10.19	4	4	8	2.212
P00338	L-lactate dehydrogenase A chain	10.24	3	3	3	3.602
P00338	L-lactate dehydrogenase A chain	7.23	2	2	2	4.610
P12318	Low affinity immunoglobulin gamma Fc region	6.94	1	1	1	0.412

	receptor II-a					
P08637	Low affinity immunoglobulin gamma Fc region receptor III-A	4.33	1	1	1	0.778
P08637	Low affinity immunoglobulin gamma Fc region receptor III-A	4.33	1	1	1	0.787
P14151	L-selectin	6.72	2	2	2	0.747
P14151	L-selectin	2.15	1	1	1	1.021
P51884	Lumican	25.44	8	8	21	1.346
P51884	Lumican	25.44	8	8	26	1.183
Q9Y5Y7	Lymphatic vessel endothelial hyaluronic acid receptor 1	2.80	1	1	2	0.995
Q9Y5Y7	Lymphatic vessel endothelial hyaluronic acid receptor 1	5.59	2	2	4	0.878
P11279	Lysosome-associated membrane glycoprotein 1	2.16	1	1	1	0.771
P11279	Lysosome-associated membrane glycoprotein 1	2.16	1	1	1	0.860
P13473	Lysosome-associated membrane glycoprotein 2	4.63	2	2	2	1.437
P13473	Lysosome-associated membrane glycoprotein 2	2.93	1	1	1	1.753
P61626	Lysozyme C	11.49	2	2	3	1.281
P61626	Lysozyme C	11.49	2	2	3	1.284
Q8N5G2	Macoilin	1.05	1	1	1	1.004
P07333	Macrophage colony-stimulating factor 1 receptor	0.93	1	1	1	0.828
P07333	Macrophage colony-stimulating factor 1 receptor	1.75	1	1	2	0.841
P22897	Macrophage mannose receptor 1	0.62	1	1	1	0.918
P14174	Macrophage migration inhibitory factor	7.83	1	1	1	1.904
Q9UEW3	Macrophage receptor MARC	1.73	1	1	1	0.483
P48740	Mannan-binding lectin serine protease 1	6.29	3	4	6	1.486
P48740	Mannan-binding lectin serine protease 1	5.29	2	3	4	0.711
O00187	Mannan-binding lectin serine protease 2	5.54	3	3	4	0.969
O00187	Mannan-binding lectin serine protease 2	7.73	4	4	4	0.784
P11226	Mannose-binding protein C	19.76	4	4	10	0.685
P11226	Mannose-binding protein C	19.76	4	4	7	0.675
P33908	Mannosyl-oligosaccharide 1,2-alpha-mannosidase IA	3.98	2	2	2	0.567
P14780	Matrix metalloproteinase-9	1.70	1	1	1	1.058
Q96HR3	Mediator of RNA polymerase II transcription subunit 30	5.06	1	1	1	0.619
Q16853	Membrane primary amine oxidase	1.57	1	1	1	1.076
Q16853	Membrane primary amine oxidase	2.88	2	2	4	1.134
P01033	Metalloproteinase inhibitor 1	5.80	1	1	1	0.642
P01033	Metalloproteinase inhibitor 1	5.80	1	1	1	0.746
P16035	Metalloproteinase inhibitor 2	4.09	1	1	1	1.080
P26038	Moesin	4.68	3	3	3	1.148
P26038	Moesin	4.51	3	3	3	2.046
P08571	Monocyte differentiation antigen CD14	18.67	6	6	11	1.209
P08571	Monocyte differentiation antigen CD14	23.47	8	8	14	1.164
Q13201	Multimerin-1	0.57	1	1	1	1.144
Q9H8L6	Multimerin-2	0.95	1	1	1	0.540
Q7Z7M0	Multiple epidermal growth factor-like domains protein 8	0.46	1	1	1	0.704

Q9UNW1	Multiple inositol polyphosphate phosphatase 1	1.85	1	1	2	0.848
Q9UNW1	Multiple inositol polyphosphate phosphatase 1	1.85	1	1	2	0.932
Q9H7P6	Multivesicular body subunit 12B	3.45	1	1	1	1.040
Q9H7P6	Multivesicular body subunit 12B	3.45	1	1	1	1.230
P05164	Myeloperoxidase	6.31	4	4	4	2.936
P05164	Myeloperoxidase	4.97	4	4	5	2.449
P35579	Myosin-9	0.51	1	1	1	1.809
P35579	Myosin-9	0.87	2	2	2	1.275
Q09013	Myotonin-protein kinase	1.27	1	1	2	
Q09013	Myotonin-protein kinase	1.27	1	1	1	
P15586	N-acetylglucosamine-6-sulfatase	1.63	1	1	1	0.288
P15586	N-acetylglucosamine-6-sulfatase	1.63	1	1	1	0.326
Q96PD5	N-acetylmuramoyl-L-alanine amidase	33.33	11	11	22	0.991
Q96PD5	N-acetylmuramoyl-L-alanine amidase	25.69	11	11	26	1.022
Q9NPH5	NADPH oxidase 4	1.38	1	1	1	1.455
Q9NPH5	NADPH oxidase 4	1.38	1	1	1	1.497
Q92859	Neogenin	0.55	1	1	1	0.999
Q92859	Neogenin	0.55	1	1	1	0.781
Q8IZJ1	Netrin receptor UNC5B	0.85	1	1	1	0.513
P13591	Neural cell adhesion molecule 1	3.15	3	3	4	0.558
P13591	Neural cell adhesion molecule 1	2.91	3	3	4	0.602
O00533	Neural cell adhesion molecule L1-like protein	1.82	2	2	3	0.811
O00533	Neural cell adhesion molecule L1-like protein	1.82	2	2	3	0.812
Q92823	Neuronal cell adhesion molecule	1.00	1	1	1	0.586
O14786	Neuropilin-1	2.71	2	2	2	0.340
O14786	Neuropilin-1	2.17	2	2	2	0.579
P59665	Neutrophil defensin 1	9.57	1	1	1	6.129
P08246	Neutrophil elastase	2.62	1	1	1	0.652
P14543	Nidogen-1	0.88	1	1	1	0.665
P14543	Nidogen-1	0.88	1	1	1	0.974
Q99784	Noelin	3.09	2	2	3	0.610
O95897	Noelin-2	2.42	1	1	1	0.643
P19838	Nuclear factor NF-kappa-B p105 subunit	1.03	1	1	4	0.440
P19838	Nuclear factor NF-kappa-B p105 subunit	1.03	1	1	6	0.510
Q9UKX7	Nuclear pore complex protein Nup50	1.71	1	1	2	1.080
Q86UD1		3.30	1	1	1	0.742
Q86UD1		9.52	2	2	2	1.085
P04746	Pancreatic alpha-amylase	2.15	1	1	1	0.799
Q86YC2	Partner and localizer of BRCA2	0.59	1	1	1	0.344
Q8WV60	Pentatricopeptide repeat-containing protein 2, mitochondrial	1.55	1	1	1	0.412
Q6UXB8	Peptidase inhibitor 16	2.16	1	1	1	1.007
P19021	Peptidyl-glycine alpha-amidating monooxygenase	0.82	1	1	1	1.097
P19021	Peptidyl-glycine alpha-amidating monooxygenase	0.82	1	1	1	1.228
P62937	Peptidyl-prolyl cis-trans isomerase A	27.88	4	4	6	1.745
P62937	Peptidyl-prolyl cis-trans isomerase A	25.45	3	4	6	1.548
P23284	Peptidyl-prolyl cis-trans isomerase B	8.80	1	2	2	1.028
Q13610	Periodic tryptophan protein 1 homolog	1.20	1	1	1	0.475
Q15063	Periostin	3.35	2	2	2	1.833
Q15063	Periostin	1.67	1	1	1	1.151
P32119	Peroxiredoxin-2	26.26	5	5	5	1.166
P32119	Peroxiredoxin-2	17.68	3	3	3	1.268

Q9NR77	Peroxisomal membrane protein 2	5.64	1	1	1	0.296
P04180	Phosphatidylcholine-sterol acyltransferase	3.18	1	1	1	0.888
P04180	Phosphatidylcholine-sterol acyltransferase	3.18	1	1	1	0.813
P80108	Phosphatidylinositol-glycan-specific phospholipase D	6.67	5	5	6	1.235
P80108	Phosphatidylinositol-glycan-specific phospholipase D	6.43	5	5	5	1.154
P00558	Phosphoglycerate kinase 1	4.08	1	1	1	4.469
P00558	Phosphoglycerate kinase 1	4.08	1	1	1	3.526
P15259	Phosphoglycerate mutase 2	4.35	1	1	1	1.821
P55058	Phospholipid transfer protein	6.69	3	3	3	0.772
P55058	Phospholipid transfer protein	10.75	4	4	4	0.855
P36955	Pigment epithelium-derived factor	38.28	15	15	34	1.131
P36955	Pigment epithelium-derived factor	33.97	13	13	38	1.036
P03952	Plasma kallikrein	18.34	11	11	13	0.926
P03952	Plasma kallikrein	19.91	13	13	17	0.999
P05155	Plasma protease C1 inhibitor	15.80	7	7	30	0.619
P05155	Plasma protease C1 inhibitor	17.60	8	8	24	0.725
P05154	Plasma serine protease inhibitor	11.33	4	4	5	0.208
P05154	Plasma serine protease inhibitor	16.01	6	6	10	0.236
P00747	Plasminogen	41.73	31	32	70	1.284
P00747	Plasminogen	40.74	30	31	70	1.314
P13796	Plastin-2	12.92	6	6	7	1.181
P13796	Plastin-2	17.38	10	10	12	1.271
P02775	Platelet basic protein	44.53	6	6	17	1.025
P02775	Platelet basic protein	36.72	5	5	13	1.053
P02776	Platelet factor 4	23.76	2	2	4	3.407
P02776	Platelet factor 4	34.65	3	3	7	2.198
P07359	Platelet glycoprotein Ib alpha chain	4.95	3	3	3	1.473
P07359	Platelet glycoprotein Ib alpha chain	6.87	4	4	5	0.886
P40197	Platelet glycoprotein V	2.86	2	2	3	1.346
P40197	Platelet glycoprotein V	3.75	2	2	2	0.931
P15151	Poliovirus receptor	1.92	1	1	1	1.607
P15151	Poliovirus receptor	3.60	2	2	3	1.380
P0CG48	Polyubiquitin-C	32.85	2	2	2	1.129
P0CG48	Polyubiquitin-C	44.67	3	3	5	1.569
P20742	Pregnancy zone protein	4.79	3	7	13	0.682
P20742	Pregnancy zone protein	3.85	2	6	12	0.532
Q9UHG3	Prenylcysteine oxidase 1	5.15	2	2	3	0.750
Q9UHG3	Prenylcysteine oxidase 1	2.97	1	1	2	0.393
Q8TBY0	Probable RNA-binding protein 46	2.25	1	1	1	0.970
Q9NQH7	Probable Xaa-Pro aminopeptidase 3	1.38	1	1	1	1.616
Q15113	Procollagen C-endopeptidase enhancer 1	3.56	1	1	2	0.560
P07737	Profilin-1	27.14	3	3	3	3.169
P07737	Profilin-1	27.14	3	3	5	4.432
P27918	Properdin	6.40	3	3	4	1.644
P27918	Properdin	3.62	2	2	3	1.582
Q8NBP7	Proprotein convertase subtilisin/kexin type 9	1.88	1	1	1	0.877
P02760	Protein AMBP	25.85	7	7	32	1.403
P02760	Protein AMBP	37.50	10	10	36	1.175
O60888	Protein CutA	7.82	1	1	1	1.109
O60888	Protein CutA	7.82	1	1	1	1.676
Q15084	Protein disulfide-isomerase A6	3.18	1	1	1	1.112
Q8WYP5	Protein ELYS	0.44	1	1	1	1.346

O95866	Protein G6b	3.73	1	1	1	0.960
Q9ULI3	Protein HEG homolog 1	1.30	1	1	1	0.377
Q9ULI3	Protein HEG homolog 1	1.81	2	2	2	0.587
P06703	Protein S100-A6	8.89	1	1	1	2.170
P06703	Protein S100-A6	8.89	1	1	2	1.929
P05109	Protein S100-A8	39.78	4	4	4	2.006
P05109	Protein S100-A8	32.26	3	3	3	1.828
P06702	Protein S100-A9	18.42	2	2	2	2.254
P06702	Protein S100-A9	17.54	2	2	2	3.214
Q9Y4B5	Protein S	0.47	1	1	2	0.802
Q9Y4B5	Protein S	0.47	1	1	1	1.321
Q9UK55	Protein Z-dependent protease inhibitor	10.14	4	4	4	1.413
Q9UK55	Protein Z-dependent protease inhibitor	10.14	4	4	4	1.757
Q92954	Proteoglycan 4	3.21	5	5	8	1.076
Q92954	Proteoglycan 4	5.56	8	8	17	1.066
P00734	Prothrombin	39.71	22	22	70	**2.003**
P00734	Prothrombin	42.77	23	23	56	**2.128**
Q9Y5H8	Protocadherin alpha-3	1.47	1	1	2	0.829
P48741	Putative heat shock 70 kDa protein 7	3.54	1	1	1	1.757
A6NL28	Putative tropomyosin alpha-3 chain-like protein	9.87	1	2	4	1.506
P14618	Pyruvate kinase PKM	4.52	2	2	2	2.318
P14618	Pyruvate kinase PKM	4.52	2	2	2	2.362
Q9Y2Q5	Ragulator complex protein LAMT	12.00	1	1	1	0.353
Q15404	Ras suppressor protein 1	2.53	1	1	1	2.000
O95294	RasGAP-activating-like protein 1	0.75	1	1	1	0.551
O00194	Ras-related protein Rab-27B	3.67	1	1	1	0.678
O00194	Ras-related protein Rab-27B	3.67	1	1	1	0.713
Q12913	Receptor-type tyrosine-protein phosphatase eta	1.94	2	2	2	0.876
Q12913	Receptor-type tyrosine-protein phosphatase eta	1.94	2	2	2	0.935
P10586	Receptor-type tyrosine-protein phosphatase F	0.52	1	1	1	1.340
P23470	Receptor-type tyrosine-protein phosphatase gamma	2.63	3	3	3	0.835
P23470	Receptor-type tyrosine-protein phosphatase gamma	2.49	3	3	3	0.827
Q86UN3	Reticulon-4 receptor-like 2	1.90	1	1	1	0.408
P00352	Retinal dehydrogenase 1	2.00	1	1	1	1.361
P00352	Retinal dehydrogenase 1	5.99	3	3	3	1.981
P02753	Retinol-binding protein 4	29.85	6	6	15	0.958
P02753	Retinol-binding protein 4	29.85	6	6	21	1.164
P52566	Rho GDP-dissociation inhibitor 2	3.48	1	1	1	1.145
Q8WZ75	Roundabout homolog 4	0.70	1	1	1	0.140
Q8WZ75	Roundabout homolog 4	2.28	2	2	2	0.358
Q59EK9	RUN domain-containing protein 3A	1.57	1	1	1	0.534
Q86VB7	Scavenger receptor cysteine-rich type 1 protein M130	2.16	2	2	2	1.119
Q86VB7	Scavenger receptor cysteine-rich type 1 protein M130	2.16	2	2	2	1.034
Q13103	Secreted phosphoprotein 24	5.69	1	1	1	1.178
P49908	Selenoprotein P	8.40	4	4	5	1.054
P49908	Selenoprotein P	4.20	2	2	4	1.508
Q96GX5	Serine/threonine-protein kinase greatwall	1.14	1	1	1	2.184

Q9P1W9	Serine/threonine-protein kinase pim-2	2.25	1	1	2	1.138
Q9P1W9	Serine/threonine-protein kinase pim-2	2.25	1	1	2	1.213
P02787	Serotransferrin	33.24	20	21	38	**0.354**
P02787	Serotransferrin	34.96	22	22	44	**0.308**
Q86U17	Serpin A11	4.03	1	1	1	0.595
P02768	Serum albumin	14.45	10	10	13	0.765
P02768	Serum albumin	11.99	8	8	14	0.787
P0DJI8	Serum amyloid A-1 protein	35.25	3	3	3	0.329
P0DJI8	Serum amyloid A-1 protein	35.25	3	3	6	0.308
P35542	Serum amyloid A-4 protein	17.69	2	2	2	0.719
P35542	Serum amyloid A-4 protein	11.54	1	1	1	1.358
P02743	Serum amyloid P-component	32.74	8	8	26	0.514
P02743	Serum amyloid P-component	32.74	8	8	33	0.647
P27169	Serum paraoxonase/arylesterase 1	23.94	7	7	9	1.446
P27169	Serum paraoxonase/arylesterase 1	25.92	8	8	11	1.745
P04278	Sex hormone-binding globulin	26.12	8	8	15	1.373
P04278	Sex hormone-binding globulin	27.11	8	8	14	1.463
Q9H299	SH3 domain-binding glutamic acid-rich-like protein 3	26.88	2	2	4	3.247
Q9H299	SH3 domain-binding glutamic acid-rich-like protein 3	26.88	2	2	4	2.136
Q9H7L9	Sin3 histone deacetylase corepressor complex component SDS3	1.83	1	1	1	0.064
Q9Y4D2	Sn1-specific diacylglycerol lipase alpha	0.96	1	1	1	0.668
Q9Y4D2	Sn1-specific diacylglycerol lipase alpha	0.96	1	1	1	0.532
A1L4H1	Soluble scavenger receptor cysteine-rich domain-containing protein SSC5D	0.95	1	1	2	0.495
A1L4H1	Soluble scavenger receptor cysteine-rich domain-containing protein SSC5D	0.95	1	1	2	0.612
P09486	SPARC	7.59	2	2	3	0.646
P09486	SPARC	10.56	3	3	4	0.733
Q14515	SPARC-like protein 1	1.51	1	1	1	
O00391	Sulfhydryl oxidase 1	7.23	4	4	5	0.778
O00391	Sulfhydryl oxidase 1	9.50	6	6	9	0.861
P00441	Superoxide dismutase [Cu-Zn]	6.49	1	1	1	1.294
P00441	Superoxide dismutase [Cu-Zn]	6.49	1	1	1	1.375
P04179	Superoxide dismutase [Mn], mitochondrial	6.31	1	1	1	0.895
Q9Y490	Talin-1	1.18	2	2	3	4.817
Q9Y490	Talin-1	2.44	5	5	5	1.762
P24821	Tenascin	0.91	2	2	2	0.974
P22105	Tenascin-X	1.96	6	6	7	0.997
P22105	Tenascin-X	2.31	8	8	10	0.889
P05452	Tetranectin	39.60	7	7	10	1.000
P05452	Tetranectin	27.23	5	5	9	1.175
P10599	Thioredoxin	12.38	1	1	1	1.254
P07996	Thrombospondin-1	15.90	17	17	26	0.945
P07996	Thrombospondin-1	17.86	17	17	21	0.842
P62328	Thymosin beta-4	15.91	1	1	1	4.825
P62328	Thymosin beta-4	15.91	1	1	1	3.476
P05543	Thyroxine-binding globulin	21.93	10	10	16	1.260
P05543	Thyroxine-binding globulin	35.42	15	15	27	1.127
P04066	Tissue alpha-L-fucosidase	2.36	1	1	1	0.683
Q8WZ42	Titin	0.03	1	1	1	0.945
Q8WZ42	Titin	0.03	1	1	1	0.900

Q9H4I3	TraB domain-containing protein	4.26	1	1	3	0.667
Q9H4I3	TraB domain-containing protein	4.26	1	1	1	1.242
Q86SZ2	Trafficking protein particle complex subunit 6B	5.70	1	1	1	0.527
Q86SZ2	Trafficking protein particle complex subunit 6B	5.70	1	1	1	0.339
P37837	Transaldolase	3.26	1	1	1	3.463
P02786	Transferrin receptor protein 1	1.84	1	1	1	0.520
P02786	Transferrin receptor protein 1	1.84	1	1	1	0.619
Q15582	Transforming growth factor-beta-induced protein ig-h3	5.12	3	3	3	1.002
Q15582	Transforming growth factor-beta-induced protein ig-h3	9.66	5	5	5	1.019
P37802	Transgelin-2	23.12	3	3	4	1.346
P37802	Transgelin-2	24.62	4	4	5	1.689
P55072	Transitional endoplasmic reticulum ATPase	2.11	1	1	1	1.742
P29401	Transketolase	1.93	1	1	1	1.176
P02766	Transthyretin	23.81	2	2	2	0.547
P02766	Transthyretin	32.65	3	3	3	0.623
Q86YW5	Trem-like transcript 1 protein	5.14	1	1	1	1.722
P06753	Tropomyosin alpha-3 chain	13.33	1	4	7	0.826
P06753	Tropomyosin alpha-3 chain	9.82	1	3	3	1.843
P67936	Tropomyosin alpha-4 chain	16.94	2	5	7	1.744
P67936	Tropomyosin alpha-4 chain	13.71	2	4	4	1.822
Q9UIG0	Tyrosine-protein kinase BAZ1B	0.61	1	1	1	0.706
P41240	Tyrosine-protein kinase CSK	2.44	1	1	1	0.228
P30530	Tyrosine-protein kinase receptor UF	1.34	1	1	1	0.923
P30530	Tyrosine-protein kinase receptor UF	1.34	1	1	1	1.046
P07911	Uromodulin	1.41	1	1	1	1.398
P06132	Uroporphyrinogen decarboxylase	2.45	1	1	2	0.363
P19320	Vascular cell adhesion protein 1	4.60	3	3	3	0.688
P19320	Vascular cell adhesion protein 1	5.14	4	4	4	0.631
P35916	Vascular endothelial growth factor receptor 3	1.54	1	1	1	0.379
P35916	Vascular endothelial growth factor receptor 3	0.73	1	1	1	0.611
Q6EMK4	Vasorin	5.35	3	3	4	0.827
Q6EMK4	Vasorin	6.39	4	4	5	0.733
P18206	Vinculin	4.50	5	5	5	2.809
P18206	Vinculin	4.06	5	5	5	3.624
P02774	Vitamin D-binding protein	45.99	21	21	91	1.231
P02774	Vitamin D-binding protein	52.53	24	24	94	1.283
P04070	Vitamin K-dependent protein C	7.38	3	3	4	0.980
P04070	Vitamin K-dependent protein C	8.03	4	4	6	0.775
P07225	Vitamin K-dependent protein S	12.43	9	9	15	0.802
P07225	Vitamin K-dependent protein S	13.02	9	9	14	0.706
P22891	Vitamin K-dependent protein Z	4.50	2	2	4	1.430
P22891	Vitamin K-dependent protein Z	6.00	3	3	3	1.077
P04004	Vitronectin	23.01	10	10	41	0.974
P04004	Vitronectin	21.97	10	10	37	0.995
P54289	Voltage-dependent calcium channel subunit alpha-2/delta-1	0.73	1	1	1	0.870
P04275	von Willebrand factor	2.31	6	6	7	0.823
P04275	von Willebrand factor	3.63	8	8	10	0.909
P12955	Xaa-Pro dipeptidase	1.42	1	1	1	0.833
Q9NQZ6	Zinc finger C4H2 domain-containing protein	3.13	1	1	2	0.226
Q9NQZ6	Zinc finger C4H2 domain-containing protein	3.13	1	1	3	0.323
Q8IWR0	Zinc finger CCCH domain-containing protein 7A	1.75	1	1	1	1.490

Q76KX8	Zinc finger protein 534	1.19	1	1	1	1.053
Q76KX8	Zinc finger protein 534	1.19	1	1	1	0.945
P25311	Zinc-alpha-2-glycoprotein	40.60	11	11	37	0.979
P25311	Zinc-alpha-2-glycoprotein	52.01	15	15	44	0.933

Table S3. A list of identified proteins (283 proteins) in two technical replicates from PQ poisoning patients and healthy controls

Accession	Description	Score	Coverage	Peptides	PSMs	Replicate 1	Replicate 2
P61981	14-3-3 protein gamma	38.5	10.93	2	3	1.06	0.96
P63104	14-3-3 protein zeta/delta	114.68	21.63	4	6	2.45	2.33
P11021	78 kDa glucose-regulated protein	38.02	7.03	3	3	0.71	0.76
Q76LX8	A disintegrin and metalloproteinase with thrombospondin motifs 13	83.44	2.31	3	4	0.89	1.22
Q13085	Acetyl-CoA carboxylase 1	0	0.3	1	1	2.05	1.84
P60709	Actin, cytoplasmic 1	274.99	26.67	9	20	2.14	2.06
Q01518	Adenylyl cyclase-associated protein 1	21.38	2.74	1	1	0.94	1.04
Q9HDC9	Adipocyte plasma membrane-associated protein	21.25	1.68	1	1	0.53	0.55
Q15848	Adiponectin	37.75	6.15	1	1	0.84	0.86
P61204	ADP-ribosylation factor 3	0	3.87	1	1	2.18	1.87
P43652	Afamin	512.51	34.06	24	60	1.11	0.96
P02763	Alpha-1-acid glycoprotein 1	50.94	15.92	6	10	0.3	0.54
P19652	Alpha-1-acid glycoprotein 2	89.17	16.42	6	10	0.33	0.31
P01011	Alpha-1-antichymotrypsin	1047.36	34.28	15	87	1.13	1.04
P01009	Alpha-1-antitrypsin	1204.08	53.83	22	102	0.3	0.31
P04217	Alpha-1B-glycoprotein	965.91	33.94	14	101	1.08	1.07
P08697	Alpha-2-antiplasmin	315.31	24.64	10	27	1.61	1.61
P02765	Alpha-2-HS-glycoprotein	1085.28	45.5	13	81	1.41	1.41
P01023	Alpha-2-macroglobulin	731.26	25.31	29	47	0.38	0.34
P12814	Alpha-actinin-1	23.68	2.58	3	3	2.89	1.42
P06733	Alpha-enolase	0	1.84	1	1	1.58	1.12
P02771	Alpha-fetoprotein	32.2	1.15	1	1	0.28	0.34
Q16706	Alpha-mannosidase 2	0	0.61	1	1	1.53	1.3
P15144	Aminopeptidase N	53.52	3.31	3	3	0.74	0.79
P03950	Angiogenin	46.78	13.61	1	1	0.56	0.6
P01019	Angiotensinogen	628.81	19.79	9	33	1.25	1.2
P01008	Antithrombin-III	1526.73	44.83	23	82	1.39	1.35
P02647	Apolipoprotein A-I	1318.38	66.29	17	110	0.31	0.3
P02652	Apolipoprotein A-II	164.93	62	5	19	0.35	0.39
P06727	Apolipoprotein A-IV	816.69	52.53	21	63	1.1	1.2
P04114	Apolipoprotein B-100	5448.5	41.62	173	498	0.9	0.94

P02654	Apolipoprotein C-I	71.01	30.12	3	4	0.57	0.5
P02655	Apolipoprotein C-II	72.51	49.5	4	6	0.57	0.58
P02656	Apolipoprotein C-III	321.22	27.27	2	10	0.59	0.63
P55056	Apolipoprotein C-IV	24.32	12.6	2	2	0.61	0.54
P05090	Apolipoprotein D	74.93	12.7	2	2	0.32	0.37
P02649	Apolipoprotein E	488.97	58.99	19	39	0.93	0.92
Q13790	Apolipoprotein F	126.84	8.28	2	3	0.52	0.62
O14791	Apolipoprotein L1	140.56	22.11	6	7	0.6	0.69
O95445	Apolipoprotein M	141.91	15.96	3	5	0.87	0.91
P08519	Apolipoprotein(a)	113.22	7.5	3	6	2.63	1.73
P17174	Aspartate aminotransferase, cytoplasmic	23.21	2.18	1	1	0.39	0.47
O75882	Attractin	177.89	8.33	9	12	0.58	0.6
P98160	Basement membrane-specific heparan sulfate proteoglycan core protein	12.32	0.48	2	2	0.9	0.44
P02749	Beta-2-glycoprotein 1	267.97	45.8	12	27	1.51	1.41
P61769	Beta-2-microglobulin	42.74	16.81	2	2	0.64	0.7
Q96KN2	Beta-Ala-His dipeptidase	38.19	10.26	5	6	0.84	1.01
P43251	Biotinidase	205.41	13.26	6	11	1.04	1.29
P13727	Bone marrow proteoglycan	72.5	8.11	1	1	1.32	0.58
P04003	C4b-binding protein alpha chain	166.61	11.89	7	13	0.32	0.34
P20851	C4b-binding protein beta chain	61.04	3.57	1	2	0.37	0.36
P12830	Cadherin-1	63.46	1.59	1	1	0.66	0.68
P55290	Cadherin-13	91.41	3.65	4	5	0.66	0.89
P33151	Cadherin-5	35.48	1.66	1	1	1.27	1.13
P00915	Carbonic anhydrase 1	109.97	9.96	3	6	1.19	1.41
Q96IY4	Carboxypeptidase B2	180.7	19.15	9	17	1.14	1.06
P15169	Carboxypeptidase N catalytic chain	120.98	18.56	8	11	0.93	1.21
P22792	Carboxypeptidase N subunit 2	147.3	23.49	11	17	1.5	1.37
Q9Y646	Carboxypeptidase Q	21.23	1.91	1	1	0.72	0.68
Q9NQ79	Cartilage acidic protein 1	66.1	5.14	2	2	0.62	0.63
P49747	Cartilage oligomeric matrix protein	40.34	6.08	4	4	0.82	0.69
P04040	Catalase	94.41	10.06	4	4	1.09	1.06
P08311	Cathepsin G	0	3.53	1	1	1.63	1.2
Q6YHK3	CD109 antigen	0	0.69	2	2	1.16	1.15
P16070	CD44 antigen	120.34	2.83	2	4	1.42	1.5

O43866	CD5 antigen-like	33.68	2.02	1	2	1.05	1.37
P43121	Cell surface glycoprotein MUC18	39.22	5.88	3	3	0.64	0.67
P00450	Ceruloplasmin	1469	38.12	35	122	1.36	1.2
P06276	Cholinesterase	183.29	15.61	8	11	1.29	1.04
Q99895	Chymotrypsin-C	23.1	2.61	1	1	0.93	0.99
P10909	Clusterin	343.62	23.83	9	23	1.44	1.24
P00740	Coagulation factor IX	32.26	6.94	4	5	1.73	2.05
P12259	Coagulation factor V	88.18	4.99	13	14	0.96	0.93
P08709	Coagulation factor VII	28.75	4.94	1	1	0.52	0.78
P00742	Coagulation factor X	158.28	16.19	6	8	1.01	1.22
P03951	Coagulation factor XI	91.59	15.04	11	15	1.4	1.31
P00748	Coagulation factor XII	155.94	19.67	13	18	1.2	0.96
P00488	Coagulation factor XIII A chain	40.66	10.38	6	6	1.31	1.74
P05160	Coagulation factor XIII B chain	172.33	16.34	9	13	1.07	0.92
P39060	Collagen alpha-1(XVIII) chain	47.71	0.97	1	1	0.87	0.91
P12111	Collagen alpha-3(VI) chain	52.24	1.57	4	4	0.76	0.77
Q9BWP8	Collectin-11	16.14	4.06	1	1	1.34	1.07
P02745	Complement C1q subcomponent subunit A	51.36	13.47	2	3	0.99	1.04
P02746	Complement C1q subcomponent subunit B	79.47	8.7	2	5	0.53	0.5
P02747	Complement C1q subcomponent subunit C	113.52	25.31	5	10	0.89	0.77
P00736	Complement C1r subcomponent	270.89	28.94	17	28	1.31	1.42
Q9NZP8	Complement C1r subcomponent-like protein	43.35	10.68	6	8	1.3	0.8
P09871	Complement C1s subcomponent	332.94	22.53	12	20	0.83	0.91
P06681	Complement C2	569.97	23.4	19	37	1.01	0.84
P01024	Complement C3	339.67	19.18	28	47	1.39	1.41
P0C0L4	Complement C4-A	2741.83	44.15	72	276	1.37	1.37
P0C0L5	Complement C4-B	2758.12	43.92	73	278	0.77	0.79
P01031	Complement C5	839.2	24.11	44	85	0.91	0.96
P13671	Complement component C6	666.93	21.73	19	41	1.43	1.2
P10643	Complement component C7	420.71	22.89	18	38	0.99	0.97
P07357	Complement component C8 alpha chain	198.74	19.52	9	14	1.72	1.5
P07358	Complement component C8 beta chain	254.26	25.38	15	27	1.05	1.09
P07360	Complement component C8 gamma chain	260.9	58.42	8	14	1.28	1.28
P02748	Complement component C9	406.33	28.98	18	43	1.36	1.38

P00751	Complement factor B	1071.04	41.88	33	95	1.3	1.24
P00746	Complement factor D	84.48	15.42	3	4	0.79	0.89
P08603	Complement factor H	1248.59	41.75	42	102	1.13	1.16
Q03591	Complement factor H-related protein 1	198.33	21.82	7	14	1.58	1.46
P36980	Complement factor H-related protein 2	152.88	23.33	6	10	1.8	1.26
P05156	Complement factor I	202.39	20.24	9	17	1.22	1.29
P08185	Corticosteroid-binding globulin	199.85	22.22	7	18	0.72	0.84
P24387	Corticotropin-releasing factor-binding protein	21.01	3.11	1	1	0.62	0.55
P02741	C-reactive protein	151.78	20.98	5	13	2.17	2.25
P01034	Cystatin-C	16.12	7.53	1	1	0.39	0.59
P54108	Cysteine-rich secretory protein 3	47.01	6.53	2	3	1.36	1.09
P81605	Dermcidin	32.69	10	1	1	0.84	0.74
Q14126	Desmoglein-2	0	0.89	1	1	1.11	1.08
Q01459	Di-N-acetylchitobiase	25.32	5.97	2	2	0.84	0.7
Q9H4A9	Dipeptidase 2	29.92	2.06	1	1	1	0.73
P27487	Dipeptidyl peptidase 4	35.36	6.79	4	4	1.17	1.46
P09172	Dopamine beta-hydroxylase	45.23	5.35	3	7	0.61	0.63
Q13822	Ectonucleotide pyrophosphatase/phosphodiesterase family member 2	25.35	2.67	1	1	0.39	0.56
Q9NZ08	Endoplasmic reticulum aminopeptidase 1	0	1.38	1	1	1.21	0.65
Q9UNN8	Endothelial protein C receptor	21.36	3.78	1	1	0.63	0.71
Q9UBQ6	Exostosin-like 2	20.54	2.12	1	1	0.94	1.02
Q16610	Extracellular matrix protein 1	109.72	17.78	7	9	1.55	1.13
P08294	Extracellular superoxide dismutase [Cu-Zn]	22.32	8.75	1	1	2.49	1
Q86UX7	Fermitin family homolog 3	82.99	6	3	4	3.1	2.19
Q9UGM5	Fetuin-B	88.23	18.32	6	9	1.22	1.13
P02671	Fibrinogen alpha chain	289.94	12.47	9	23	0.88	1.15
P02675	Fibrinogen beta chain	36.45	6.92	6	7	1.17	0.88
P02679	Fibrinogen gamma chain	14.13	1.99	1	1	0.8	0.95
P02751	Fibronectin	288.05	8.21	15	21	1.04	1.32
P23142	Fibulin-1	45.2	6.97	4	6	1.23	1.17
Q15485	Ficolin-2	21.23	5.11	1	2	0.6	0.64
O75636	Ficolin-3	293.58	34.11	10	24	0.92	0.92
P30043	Flavin reductase (NADPH)	60.24	7.28	1	2	1.85	0.82
P04075	Fructose-bisphosphate aldolase A	17.56	2.2	3	3	1.81	1.97

P05062	Fructose-bisphosphate aldolase B	70.31	7.14	2	2	1.3	1.31
P16930	Fumarylacetoacetase	39.32	3.1	4	4	0.66	1.61
Q08380	Galectin-3-binding protein	57.21	10.26	5	7	0.87	1.07
Q92820	Gamma-glutamyl hydrolase	88.42	9.12	2	3	1.21	0.78
P06396	Gelsolin	560.72	32.86	23	43	1.02	1.01
P22352	Glutathione peroxidase 3	79.27	15.93	3	6	0.84	0.76
P68871	Hemoglobin subunit beta	54.6	27.89	4	7	0.63	0.64
P02790	Hemopexin	1695.47	52.81	23	134	0.93	0.86
P05546	Heparin cofactor 2	305.65	18.24	15	42	1.18	1.14
Q04756	Hepatocyte growth factor activator	154.4	9.01	5	7	1.35	1.44
P26927	Hepatocyte growth factor-like protein	99.88	5.63	4	5	1.33	1.09
P04196	Histidine-rich glycoprotein	401.29	25.52	14	41	0.67	0.69
P0C0S5	Histone H2A.Z	0	5.47	1	1	2.06	2.06
Q14520	Hyaluronan-binding protein 2	65.4	9.11	6	7	0.71	0.84
P01834	Ig kappa chain C region	47.57	15.09	1	1	0.32	0.39
P0CG05	Ig lambda-2 chain C regions	22.94	9.43	1	1	0.49	0.44
Q9Y6R7	IgGFc-binding protein	26.69	1.22	3	3	0.68	0.63
P17936	Insulin-like growth factor-binding protein 3	87.33	19.93	5	7	1.63	1.36
P35858	Insulin-like growth factor-binding protein complex acid labile subunit	986.75	37.85	19	49	1.07	1.19
P19827	Inter-alpha-trypsin inhibitor heavy chain H1	986.72	23.16	18	84	0.99	1.06
P19823	Inter-alpha-trypsin inhibitor heavy chain H2	1608.54	27.06	27	127	1.15	1.23
Q06033	Inter-alpha-trypsin inhibitor heavy chain H3	276.42	10.34	8	16	0.91	1.18
Q14624	Inter-alpha-trypsin inhibitor heavy chain H4	1348.2	39.03	30	98	1.21	1.19
P13598	Intercellular adhesion molecule 2	0	2.55	1	1	1.57	1.64
P32942	Intercellular adhesion molecule 3	0	1.65	1	1	1.36	1.17
Q9NPH3	Interleukin-1 receptor accessory protein	30.23	6.32	4	6	0.75	0.7
O75874	Isocitrate dehydrogenase [NADP] cytoplasmic	25.13	2.17	1	1	1.99	2.33
P29622	Kallistatin	266.44	32.79	15	26	1.08	1.03
P04264	Keratin, type II cytoskeletal 1	32.12	1.4	1	1	0.4	0.59
P01042	Kininogen-1	718.96	28.42	20	77	1.06	1.03
P02788	Lactotransferrin	33.69	3.24	6	7	1.75	1.74
P02750	Leucine-rich alpha-2-glycoprotein	346.19	44.09	13	37	1.25	1.24
P30740	Leukocyte elastase inhibitor	38.88	3.17	1	1	1.05	0.67
P18428	Lipopolysaccharide-binding protein	182.66	17.26	5	9	2.23	2.21

P00338	L-lactate dehydrogenase A chain	28.16	10.24	3	3	3.6	4.61
P08637	Low affinity immunoglobulin gamma Fc region receptor III-A	21.05	4.33	1	1	0.78	0.79
P14151	L-selectin	23.54	6.72	2	2	0.75	1.02
P51884	Lumican	350.68	25.44	8	26	1.18	1.35
Q9Y5Y7	Lymphatic vessel endothelial hyaluronic acid receptor 1	31.03	2.8	1	2	1	0.88
P11279	Lysosome-associated membrane glycoprotein 1	25.3	2.16	1	1	0.77	0.86
P13473	Lysosome-associated membrane glycoprotein 2	23.99	4.63	1	1	1.75	1.44
P61626	Lysozyme C	32.88	11.49	2	3	1.28	1.28
P07333	Macrophage colony-stimulating factor 1 receptor	35.68	0.93	1	2	0.84	0.83
P48740	Mannan-binding lectin serine protease 1	96.48	6.29	4	6	1.49	0.71
O00187	Mannan-binding lectin serine protease 2	47.46	5.54	4	4	0.97	0.78
P11226	Mannose-binding protein C	187	19.76	4	10	0.68	0.68
Q16853	Membrane primary amine oxidase	26.09	1.57	2	4	1.13	1.08
P01033	Metalloproteinase inhibitor 1	44.59	5.8	1	1	0.64	0.75
P26038	Moesin	31.43	4.68	3	3	2.05	1.15
P08571	Monocyte differentiation antigen CD14	194.64	18.67	8	14	1.16	1.21
Q9UNW1	Multiple inositol polyphosphate phosphatase 1	41.61	1.85	1	2	0.85	0.93
Q9H7P6	Multivesicular body subunit 12B	32.23	3.45	1	1	1.04	1.23
P05164	Myeloperoxidase	47.86	6.31	4	4	2.94	2.45
P35579	Myosin-9	0	0.51	1	1	1.81	1.27
P15586	N-acetylglucosamine-6-sulfatase	14.79	1.63	1	1	0.29	0.33
Q96PD5	N-acetylmuramoyl-L-alanine amidase OS	339.87	33.33	11	22	0.99	1.02
Q9NPH5	NADPH oxidase 4	0	1.38	1	1	1.46	1.5
Q92859	Neogenin	27.38	0.55	1	1	1	0.78
P13591	Neural cell adhesion molecule 1	30.18	3.15	3	4	0.56	0.6
O00533	Neural cell adhesion molecule L1-like protein	26.22	1.82	2	3	0.81	0.81
O14786	Neuropilin-1	46.91	2.71	2	2	0.34	0.58
P14543	Nidogen-1	20.39	0.88	1	1	0.97	0.67
P19838	Nuclear factor NF-kappa-B p105 subunit	34.46	1.03	1	4	0.44	0.51
Q86UD1	Out at first protein homolog	21.54	3.3	1	1	0.74	1.09
P19021	Peptidyl-glycine alpha-amidating monooxygenase	0	0.82	1	1	1.1	1.23
P62937	Peptidyl-prolyl cis-trans isomerase A	67.32	27.88	4	6	1.74	1.55
Q15063	Periostin	34.99	3.35	2	2	1.83	1.15
P32119	Peroxiredoxin-2	59.65	26.26	3	3	1.27	1.17

P04180	Phosphatidylcholine-sterol acyltransferase	32.48	3.18	1	1	0.81	0.89
P80108	Phosphatidylinositol-glycan-specific phospholipase D	78.2	6.67	5	6	1.23	1.15
P00558	Phosphoglycerate kinase 1	98.25	4.08	1	1	4.47	3.53
P55058	Phospholipid transfer protein	44.14	6.69	4	4	0.85	0.77
P36955	Pigment epithelium-derived factor	386.59	38.28	15	34	1.13	1.04
P03952	Plasma kallikrein	143.63	18.34	13	17	1	0.93
P05155	Plasma protease C1 inhibitor	264.61	15.8	8	24	0.72	0.62
P05154	Plasma serine protease inhibitor	98.05	11.33	6	10	0.24	0.21
P00747	Plasminogen	1058.05	41.73	32	70	1.28	1.31
P13796	Plastin-2	101.4	12.92	6	7	1.18	1.27
P02775	Platelet basic protein	183.85	44.53	6	17	1.02	1.05
P02776	Platelet factor 4	58.45	23.76	3	7	2.2	3.41
P07359	Platelet glycoprotein Ib alpha chain	46.94	4.95	3	3	1.47	0.89
P40197	Platelet glycoprotein V	28.79	2.86	2	2	0.93	1.35
P15151	Poliovirus receptor	41.51	1.92	1	1	1.61	1.38
P0CG48	Polyubiquitin-C	30.62	32.85	3	5	1.57	1.13
P20742	Pregnancy zone protein	172.95	4.79	6	12	0.53	0.68
Q9UHG3	Prenylcysteine oxidase 1	70.39	5.15	2	3	0.75	0.39
P07737	Profilin-1	38.39	27.14	3	5	4.43	3.17
P27918	Properdin	57.83	6.4	3	4	1.64	1.58
P02760	Protein AMBP	537.05	25.85	10	36	1.17	1.4
O60888	Protein CutA	41.26	7.82	1	1	1.11	1.68
Q9ULI3	Protein HEG homolog 1	43.86	1.3	1	1	0.38	0.59
P06703	Protein S100-A6	0	8.89	1	1	2.17	1.93
P05109	Protein S100-A8	52.08	39.78	4	4	2.01	1.83
P06702	Protein S100-A9	29.69	18.42	2	2	3.21	2.25
Q9Y4B5	Protein SOGA2	15.23	0.47	1	2	0.8	1.32
Q9UK55	Protein Z-dependent protease inhibitor	101.25	10.14	4	4	1.41	1.76
Q92954	Proteoglycan 4	60.24	3.21	5	8	1.08	1.07
P00734	Prothrombin	1041.07	39.71	22	70	2	2.13
P14618	Pyruvate kinase PKM	24.97	4.52	2	2	2.36	2.32
O00194	Ras-related protein Rab-27B	24.57	3.67	1	1	0.68	0.71
Q12913	Receptor-type tyrosine-protein phosphatase eta	45.6	1.94	2	2	0.88	0.94
P23470	Receptor-type tyrosine-protein phosphatase gamma	40.44	2.63	3	3	0.84	0.83

P00352	Retinal dehydrogenase 1	0	2	3	3	1.98	1.36
P02753	Retinol-binding protein 4	146.89	29.85	6	21	1.16	0.96
Q8WZ75	Roundabout homolog 4	18.41	0.7	1	1	0.14	0.36
Q86VB7	Scavenger receptor cysteine-rich type 1 protein M130	49.91	2.16	2	2	1.12	1.03
P49908	Selenoprotein P	19.64	8.4	2	4	1.51	1.05
Q9P1W9	Serine/threonine-protein kinase pim-2	0	2.25	1	2	1.14	1.21
P02787	Serotransferrin	481.09	33.24	22	44	0.31	0.35
P02768	Serum albumin	169.97	14.45	10	13	0.26	0.29
P0DJI8	Serum amyloid A-1 protein	93.65	35.25	3	3	0.33	0.31
P35542	Serum amyloid A-4 protein	23.46	17.69	2	2	0.72	1.36
P02743	Serum amyloid P-component	265.56	32.74	10	13	0.51	0.65
P27169	Serum paraoxonase/arylesterase 1	104.36	23.94	7	9	1.45	1.75
P04278	Sex hormone-binding globulin	261.91	26.12	8	15	1.37	1.46
Q9H299	SH3 domain-binding glutamic acid-rich-like protein 3	80.68	26.88	2	4	3.25	2.14
Q9Y4D2	Sn1-specific diacylglycerol lipase alpha	26.35	0.96	1	1	0.67	0.53
A1L4H1	Soluble scavenger receptor cysteine-rich domain-containing protein SSC5D	83.06	0.95	1	2	0.5	0.61
P09486	SPARC	91.15	7.59	2	3	0.65	0.73
O00391	Sulfhydryl oxidase 1	129.14	7.23	6	9	0.78	0.86
P00441	Superoxide dismutase [Cu-Zn]	48.3	6.49	1	1	1.29	1.38
Q9Y490	Talin-1	66.06	1.18	2	3	4.82	1.76
P22105	Tenascin-X	97.65	1.96	6	7	1	0.89
P05452	Tetranectin	198.14	39.6	5	9	1.17	1
P07996	Thrombospondin-1	354.62	15.9	17	26	0.95	0.84
P62328	Thymosin beta-4	29.05	15.91	1	1	4.82	3.48
P05543	Thyroxine-binding globulin	180.89	21.93	15	27	1.13	1.26
Q8WZ42	Titin	12.32	0.03	1	1	0.94	0.9
Q9H4I3	TraB domain-containing protein	24.35	4.26	1	3	0.67	1.24
Q86SZ2	Trafficking protein particle complex subunit 6B	43.84	5.7	1	1	0.34	0.53
P02786	Transferrin receptor protein 1	30.47	1.84	1	1	0.62	0.52
Q15582	Transforming growth factor-beta-induced protein ig-h3	58.68	5.12	5	5	1.02	1
P37802	Transgelin-2	42.94	23.12	4	5	1.69	1.35
P02766	Transthyretin	37.18	23.81	3	3	0.62	0.55
P06753	Tropomyosin alpha-3 chain	24.42	13.33	4	7	1.84	0.83
P67936	Tropomyosin alpha-4 chain	26.87	16.94	5	7	1.74	1.82

P30530	Tyrosine-protein kinase receptor UFO	43.74	1.34	1	1	0.92	1.05
P19320	Vascular cell adhesion protein 1	63.88	4.6	3	3	0.69	0.63
P35916	Vascular endothelial growth factor receptor 3	53.23	1.54	1	1	0.38	0.61
Q6EMK4	Vasorin	56.66	5.35	4	5	0.73	0.83
P18206	Vinculin	35.97	4.5	5	5	3.62	2.81
P02774	Vitamin D-binding protein	759.28	45.99	21	91	1.23	1.28
P04070	Vitamin K-dependent protein C	86.15	7.38	4	6	0.77	0.98
P07225	Vitamin K-dependent protein S	150.45	12.43	9	14	0.71	0.8
P22891	Vitamin K-dependent protein Z	79.45	4.5	2	4	1.43	1.08
P04004	Vitronectin	577.13	23.01	10	37	0.99	0.97
P04275	von Willebrand factor	51.1	2.31	8	10	0.91	0.82
Q9NQZ6	Zinc finger C4H2 domain-containing protein	31.26	3.13	1	2	0.23	0.32
Q76KX8	Zinc finger protein 534	21.69	1.19	1	1	1.05	0.94
P25311	Zinc-alpha-2-glycoprotein	551.69	40.6	11	37	0.98	0.93

Table S4. A list of differentially expressed proteins (81 proteins) in two technical replicates from PQ poisoning patients and healthy controls

**Accession**	**Description**	**Score**	**Coverage**	**Peptides**	**PSMs**	**Replication 1**	**Repliaction 2**
P63104	14-3-3 protein zeta/delta	114.68	21.63	4	6	2.45	2.33
Q13085	Acetyl-CoA carboxylase 1	0.00	0.30	1	1	2.05	1.84
P60709	Actin, cytoplasmic 1	274.99	26.67	9	20	2.14	2.06
Q9HDC9	Adipocyte plasma membrane-associated protein	0.00	1.68	1	1	0.53	0.55
P61204	ADP-ribosylation factor 3	0.00	3.87	1	1	2.18	1.87
P02763	Alpha-1-acid glycoprotein 1	50.94	15.92	6	10	0.30	0.54
P19652	Alpha-1-acid glycoprotein 2	89.17	16.42	6	10	0.33	0.31
P01009	Alpha-1-antitrypsin	1204.08	53.83	22	102	0.30	0.31
P08697	Alpha-2-antiplasmin	315.31	24.64	10	27	1.61	1.61
P01023	Alpha-2-macroglobulin	731.26	25.31	29	47	0.38	0.34
P02771	Alpha-fetoprotein	0.00	1.15	1	1	0.28	0.34
P03950	Angiogenin	46.78	13.61	1	1	0.56	0.60
P02647	Apolipoprotein A-I	1318.38	66.29	17	110	0.31	0.30

P02652	Apolipoprotein A-II	164.93	62.00	5	19	0.35	0.39
P02654	Apolipoprotein C-I	71.01	30.12	3	4	0.57	0.50
P02655	Apolipoprotein C-II	72.51	49.50	4	6	0.57	0.58
P02656	Apolipoprotein C-III	321.22	27.27	2	10	0.59	0.63
P55056	Apolipoprotein C-IV	0.00	12.60	2	2	0.61	0.54
P05090	Apolipoprotein D	74.93	12.70	2	2	0.32	0.37
Q13790	Apolipoprotein F	126.84	8.28	2	3	0.52	0.62
O14791	Apolipoprotein L1	140.56	22.11	6	7	0.60	0.69
P17174	Aspartate aminotransferase, cytoplasmic	0.00	2.18	1	1	0.39	0.47
O75882	Attractin	177.89	8.33	9	12	0.58	0.60
P04003	C4b-binding protein alpha chain	166.61	11.89	7	13	0.32	0.34
P20851	C4b-binding protein beta chain	61.04	3.57	1	2	0.37	0.36
Q9NQ79	Cartilage acidic protein 1	66.10	5.14	2	2	0.62	0.63
P43121	Cell surface glycoprotein MUC18	39.22	5.88	3	3	0.64	0.67
P00740	Coagulation factor IX	32.26	6.94	4	5	1.73	2.05
P02746	Complement C1q subcomponent subunit B	79.47	8.70	2	5	0.53	0.50
P07357	Complement component C8 alpha chain	198.74	19.52	9	14	1.72	1.50
Q03591	Complement factor H-related protein 1	198.33	21.82	7	14	1.58	1.46
P24387	Corticotropin-releasing factor-binding protein	0.00	3.11	1	1	0.62	0.55
P02741	C-reactive protein	151.78	20.98	5	13	2.17	2.25
P01034	Cystatin-C	16.12	7.53	1	1	0.39	0.59
P09172	Dopamine beta-hydroxylase	45.23	5.35	3	7	0.61	0.63
Q13822	Ectonucleotide pyrophosphatase/phosphodiesterase family member 2	0.00	2.67	1	1	0.39	0.56
Q15485	Ficolin-2	0.00	5.11	1	2	0.60	0.64
P04075	Fructose-bisphosphate aldolase A	17.56	2.20	3	3	1.81	1.97
P68871	Hemoglobin subunit beta	54.60	27.89	4	7	0.63	0.64
P0C0S5	Histone H2A.Z	0.00	5.47	1	1	2.06	2.06
P01834	Ig kappa chain C region	47.57	15.09	1	1	0.32	0.39
P0CG05	Ig lambda-2 chain C regions	22.94	9.43	1	1	0.49	0.44
Q9Y6R7	IgGFc-binding protein OS=Homo sapiens GN=FCGBP PE=1 SV=3 - [FCGBP_HUMAN]	26.68810198	1.22	1	3	0.68	0.56
P13598	Intercellular adhesion molecule 2	0.00	2.55	1	1	1.57	1.64
O75874	Isocitrate dehydrogenase [NADP] cytoplasmic	25.13	2.17	1	1	1.99	2.33
P04264	Keratin, type II cytoskeletal 1	0.00	1.40	1	1	0.40	0.59

P02788	Lactotransferrin	33.69	3.24	6	7	1.75	1.74
P18428	Lipopolysaccharide-binding protein	182.66	17.26	5	9	2.23	2.21
P00338	L-lactate dehydrogenase A chain	28.16	10.24	3	3	3.60	4.61
P05164	Myeloperoxidase	47.86	6.31	4	4	2.94	2.45
P15586	N-acetylglucosamine-6-sulfatase	14.79	1.63	1	1	0.29	0.33
P13591	Neural cell adhesion molecule 1	30.18	3.15	3	4	0.56	0.60
O14786	Neuropilin-1	46.91	2.71	2	2	0.34	0.58
P19838	Nuclear factor NF-kappa-B p105 subunit	34.46	1.03	1	4	0.44	0.51
P62937	Peptidyl-prolyl cis-trans isomerase A	67.32	27.88	4	6	1.74	1.55
P00558	Phosphoglycerate kinase 1	98.25	4.08	1	1	4.47	3.53
P05154	Plasma serine protease inhibitor	98.05	11.33	6	10	0.24	0.21
P0CG48	Polyubiquitin-C	30.62	32.85	3	5	1.57	1.13
P20742	Pregnancy zone protein	172.95	4.79	6	12	0.53	0.68
P07737	Profilin-1	38.39	27.14	3	5	4.43	3.17
P27918	Properdin	57.83	6.40	3	4	1.64	1.58
Q9ULI3	Protein HEG homolog 1	43.86	1.30	1	1	0.38	0.59
P06703	Protein S100-A6	0.00	8.89	1	1	2.17	1.93
P05109	Protein S100-A8	52.08	39.78	4	4	2.01	1.83
P06702	Protein S100-A9	29.69	18.42	2	2	3.21	2.25
P00734	Prothrombin	1041.07	39.71	22	70	2.00	2.13
P14618	Pyruvate kinase PKM	24.97	4.52	2	2	2.36	2.32
Q8WZ75	Roundabout homolog 4	18.41	0.70	1	1	0.14	0.36
P02787	Serotransferrin	481.09	33.24	22	44	0.31	0.35
P02743	Serum amyloid P-component	265.56	32.74	10	13	0.51	0.65
P0DJI8	Serum amyloid A-1 protein	93.65	35.25	3	3	0.33	0.31
Q9Y4D2	Sn1-specific diacylglycerol lipase alpha	28.43	0.96	1	1	0.53	0.66
A1L4H1	Soluble scavenger receptor cysteine-rich domain-containing protein SSC5D	83.06	0.95	1	2	0.50	0.61
P62328	Thymosin beta-4	29.05	15.91	1	1	4.82	3.48
Q86SZ2	Trafficking protein particle complex subunit 6B	0.00	5.70	1	1	0.34	0.53
P02786	Transferrin receptor protein 1	30.47	1.84	1	1	0.62	0.52
P02766	Transthyretin	37.18	23.81	3	3	0.62	0.55
P67936	Tropomyosin alpha-4 chain	26.87	16.94	5	7	1.74	1.82
P35916	Vascular endothelial growth factor receptor 3	0.00	1.54	1	1	0.38	0.61

P18206	Vinculin	35.97	4.50	5	5	3.62	2.81
Q9NQZ6	Zinc finger C4H2 domain-containing protein	31.26	3.13	1	2	0.23	0.32

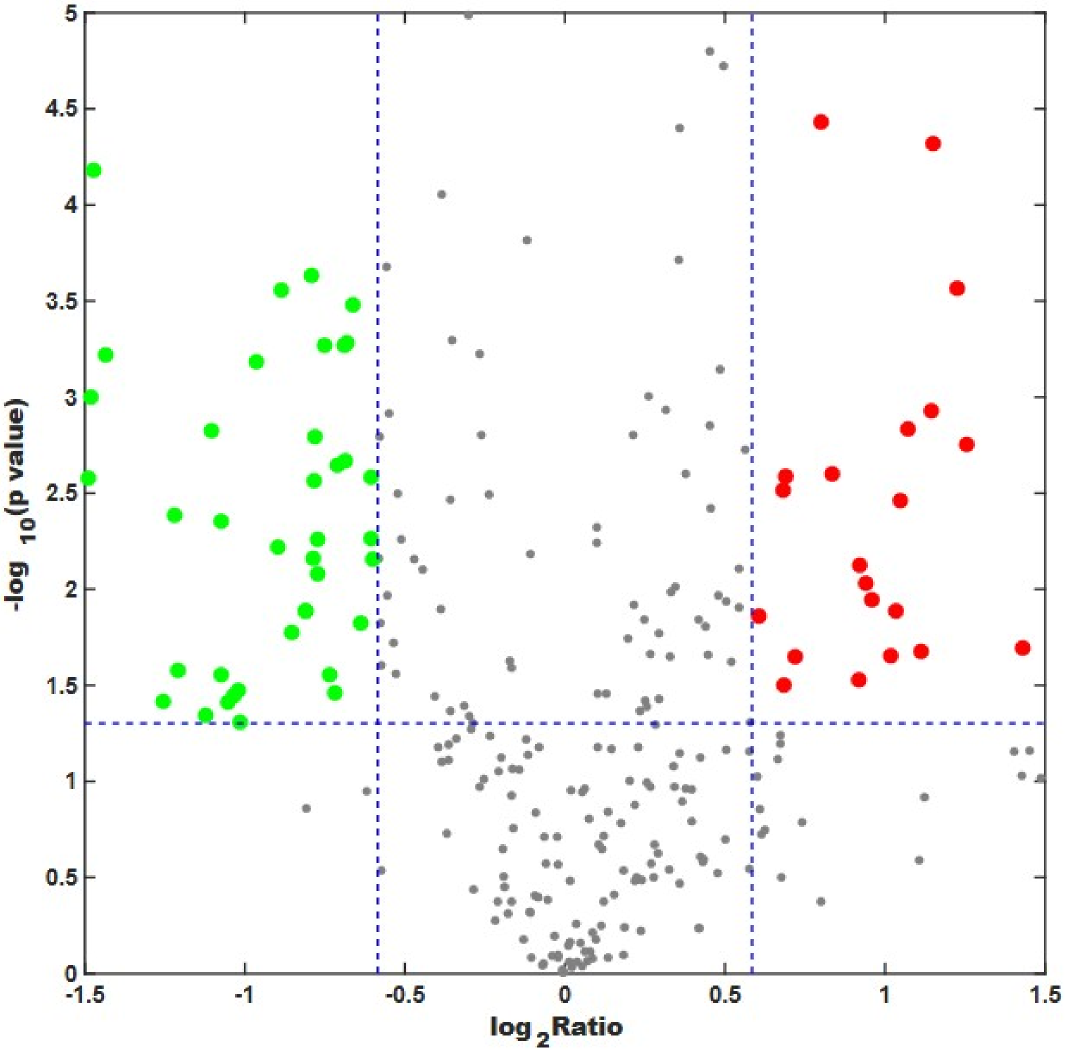

Fig. S2. The horizontal coordinates represent the proteins with a log_2^{Ratio} (PQ intoxication/Control)>0.58 r <-0.58 (fold changes >1.5 are shown in red and 0.667 are shown in green). The vertical axis is the -log_{10}(*p*-value)>1.30 (average *p*-value <0.05) of differential expression proteins in two technical replicates. Every point represents an individual protein. (For interpretation of the references to colour in this figure legend, the reader is referred to the web version of this article.)

Table S5. The enriched pathways among the differentially expressed proteins

Ingenuity Canonical Pathways	-log(p-value)	Ratio	Molecules
LXR/RXR Activation	28.3	0.174	TTR,APOF,APOC4,APOA2,APOC2,NFKB1,SERPINF2,APOL1,ALB,APOA1,TF,ORM1,SAA1,APOC1,ORM2,ACACA,SERPINA1,S100A8,LBP,APOD,APOC3
FXR/RXR Activation	21	0.136	TTR,APOF,APOC4,APOA2,APOC2,SERPINF2,APOL1,ALB,APOA1,TF,ORM1,SAA1,APOC1,ORM2,SERPINA1,APOD,APOC3
Atherosclerosis Signaling	19.3	0.129	VCAM1,APOF,APOC4,APOA2,APOC2,NFKB1,APOL1,ALB,APOA1,ORM1,APOC1,ORM2,SERPINA1,S100A8,APOD,APOC3
Clathrin-mediated Endocytosis Signaling	19	0.0909	APOF,APOC4,ACTB,APOA2,APOC2,F2,APOL1,ALB,APOA1,TF,ORM1,APOC1,TFRC,ORM2,SERPINA1,S100A8,APOD,APOC3
Acute Phase Response Signaling	18.7	0.101	TTR,C4BPB,APOA2,NFKB1,F2,SERPINF2,ALB,APOA1,TF,ORM1,C4BPA,SAA1,CRP,ORM2,SERPINA1,LBP,A2M
IL-12 Signaling and Production in Macrophages	16.7	0.104	APOF,APOC4,APOA2,APOC2,NFKB1,APOL1,ALB,APOA1,ORM1,APOC1,ORM2,S100A8,SERPINA1,APOD,APOC3
Production of Nitric Oxide and Reactive Oxygen Species in Macrophages	16.2	0.0829	APOF,APOC4,APOA2,APOC2,NFKB1,APOL1,ALB,MPO,APOA1,ORM1,APOC1,ORM2,SERPINA1,S100A8,APOD,APOC3
Coagulation System	8.25	0.171	F9,SERPINA5,SERPINA1,A2M,F2,SERPINF2
Complement System	4.86	0.111	C4BPB,C4BPA,C8A,C1QB
Glycolysis I	3.91	0.125	PGK1,PKM,ALDOA
Actin Cytoskeleton Signaling	3.54	0.0271	PFN1,ACTB,LBP,VCL,TMSB10/TMSB4X,F2
Hepatic Fibrosis / Hepatic Stellate Cell Activation	3.04	0.0273	VCAM1,FLT4,LBP,NFKB1,A2M
Role of IL-17A in Psoriasis	2.91	0.154	S100A9,S100A8
IL-6 Signaling	2.73	0.0312	CRP,LBP,NFKB1,A2M
Melatonin Degradation III	2.39	1	MPO
Gluconeogenesis I	2.33	0.08	PGK1,ALDOA
VEGF Signaling	2.1	0.03	ACTB,FLT4,VCL
ILK Signaling	2.1	0.0207	ACTB,VCL,TMSB10/TMSB4X,NFKB1
L-cysteine	2.09	0.5	GOT1

Degradation III			
IL-8 Signaling	2.07	0.0204	VCAM1,MPO,FLT4,NFKB1
LPS/IL-1 Mediated Inhibition of RXR Function	1.98	0.0191	APOC4,APOC1,APOC2,LBP
Integrin Signaling	1.96	0.0189	PFN1,ARF3,ACTB,VCL
Glucocorticoid Receptor Signaling	1.92	0.0149	VCAM1,ACTB,KRT1,NFKB1,A2M
Intrinsic Prothrombin Activation Pathway	1.92	0.0488	F9,F2
Biotin-carboxyl Carrier Protein Assembly	1.91	0.333	ACACA
Glutamate Degradation II	1.91	0.333	GOT1
Aspartate Biosynthesis	1.91	0.333	GOT1
iNOS Signaling	1.86	0.0455	LBP,NFKB1
Catecholamine Biosynthesis	1.79	0.25	DBH
L-cysteine Degradation I	1.79	0.25	GOT1
Pyruvate Fermentation to Lactate	1.69	0.2	LDHA
Aspartate Degradation II	1.55	0.143	GOT1
Remodeling of Epithelial Adherens Junctions	1.53	0.0303	ACTB,VCL
Tight Junction Signaling	1.52	0.0181	ACTB,VCL,NFKB1
Lymphotoxin β Receptor Signaling	1.51	0.0299	VCAM1,NFKB1
PPARα/RXRα Activation	1.5	0.0179	APOA1,APOA2,NFKB1
Germ Cell-Sertoli Cell	1.5	0.0178	ACTB,VCL,A2M

Junction Signaling			
Sucrose Degradation V (Mammalian)	1.49	0.125	ALDOA
IL-10 Signaling	1.49	0.029	LBP,NFKB1
TREM1 Signaling	1.48	0.0286	MPO,NFKB1
Sertoli Cell-Sertoli Cell Junction Signaling	1.47	0.0173	ACTB,VCL,A2M
Caveolar-mediated Endocytosis Signaling	1.47	0.0282	ALB,ACTB
Agranulocyte Adhesion and Diapedesis	1.45	0.017	VCAM1,ICAM2,ACTB
Toll-like Receptor Signaling	1.42	0.0267	LBP,NFKB1
IL-15 Signaling	1.41	0.0263	VCAM1,NFKB1
IL-17 Signaling	1.33	0.0235	CRP,NFKB1
Regulation of Actin-based Motility by Rho	1.32	0.0233	PFN1,ACTB
LPS-stimulated MAPK Signaling	1.31	0.023	LBP,NFKB1
VEGF Family Ligand-Receptor Interactions	1.3	0.0227	FLT4,NRP1
Crosstalk between Dendritic Cells and Natural Killer Cells	1.29	0.0225	ACTB,NFKB1
Leukocyte Extravasation Signaling	1.28	0.0146	VCAM1,ACTB,VCL
Osteoarthritis Pathway	1.28	0.0146	S100A9,S100A8,NFKB1
Acute Myeloid Leukemia Signaling	1.27	0.0217	NFKB1,IDH1
Death Receptor Signaling	1.27	0.0217	ACTB,NFKB1

Phenylalanine Degradation IV (Mammalian, via Side Chain)	1.25	0.0714	GOT1
FAK Signaling	1.22	0.0204	ACTB,VCL
Extrinsic Prothrombin Activation Pathway	1.2	0.0625	F2
Virus Entry via Endocytic Pathways	1.19	0.0196	ACTB,TFRC
Paxillin Signaling	1.13	0.0182	ACTB,VCL
Inflammasome pathway	1.11	0.05	NFKB1
PTEN Signaling	1.07	0.0168	FLT4,NFKB1
RhoA Signaling	1.05	0.0164	PFN1,ACTB
PI3K/AKT Signaling	1.05	0.0163	YWHAZ,NFKB1
Role of JAK1, JAK2 and TYK2 in Interferon Signaling	1.03	0.0417	NFKB1
IL-17A Signaling in Gastric Cells	1.01	0.04	NFKB1
Role of Pattern Recognition Receptors in Recognition of Bacteria and Viruses	1	0.0153	C1QB,NFKB1
HMGB1 Signaling	1	0.0153	VCAM1,NFKB1
p70S6K Signaling	1	0.0153	YWHAZ,F2
Gα12/13 Signaling	0.979	0.0148	NFKB1,F2
IL-15 Production	0.966	0.0357	NFKB1
Sirtuin Signaling Pathway	0.963	0.0106	PGK1,LDHA,NFKB1
TNFR2 Signaling	0.952	0.0345	NFKB1
Epithelial Adherens Junction Signaling	0.937	0.014	ACTB,VCL
4-1BB Signaling in T Lymphocytes	0.911	0.0312	NFKB1

Neuroinflammation Signaling Pathway	0.904	0.00997	VCAM1,CRP,NFKB1
Type II Diabetes Mellitus Signaling	0.894	0.0132	PKM,NFKB1
TWEAK Signaling	0.887	0.0294	NFKB1
MIF-mediated Glucocorticoid Regulation	0.875	0.0286	NFKB1
IL-17A Signaling in Fibroblasts	0.875	0.0286	NFKB1
Superpathway of Methionine Degradation	0.875	0.0286	GOT1
Hepatic Cholestasis	0.867	0.0127	LBP,NFKB1
Inhibition of Matrix Metalloproteases	0.842	0.0263	A2M
Granulocyte Adhesion and Diapedesis	0.832	0.012	VCAM1,ICAM2
April Mediated Signaling	0.831	0.0256	NFKB1
Tec Kinase Signaling	0.824	0.0119	ACTB,NFKB1
Triacylglycerol Degradation	0.821	0.025	DAGLA
Role of PKR in Interferon Induction and Antiviral Response	0.811	0.0244	NFKB1
B Cell Activating Factor Signaling	0.811	0.0244	NFKB1
Mechanisms of Viral Exit from Host Cells	0.811	0.0244	ACTB
Role of RIG1-like Receptors in Antiviral Innate Immunity	0.802	0.0238	NFKB1
Role of IL-17F in Allergic	0.802	0.0238	NFKB1

Inflammatory Airway Diseases			
MIF Regulation of Innate Immunity	0.792	0.0233	NFKB1
NF-κB Signaling	0.785	0.0112	FLT4,NFKB1
IL-9 Signaling	0.774	0.0222	NFKB1
RAR Activation	0.752	0.0107	ACTB,NFKB1
TNFR1 Signaling	0.749	0.0208	NFKB1
Cell Cycle: G2/M DNA Damage Checkpoint Regulation	0.741	0.0204	YWHAZ
Cancer Drug Resistance By Drug Efflux	0.741	0.0204	NFKB1
CD27 Signaling in Lymphocytes	0.718	0.0192	NFKB1
Semaphorin Signaling in Neurons	0.718	0.0192	NRP1
Thrombin Signaling	0.704	0.00995	NFKB1,F2
Regulation of Cellular Mechanics by Calpain Protease	0.696	0.0182	VCL
OX40 Signaling Pathway	0.689	0.0179	NFKB1
Induction of Apoptosis by HIV1	0.662	0.0167	NFKB1
PCP pathway	0.662	0.0167	PFN1
AMPK Signaling	0.66	0.0093	ACTB,ACACA
Activation of IRF by Cytosolic Pattern Recognition Receptors	0.656	0.0164	NFKB1
Superpathway of Melatonin Degradation	0.656	0.0164	MPO
Wnt/Ca+ pathway	0.65	0.0161	NFKB1

ERK5 Signaling	0.626	0.0152	YWHAZ
Agrin Interactions at Neuromuscular Junction	0.62	0.0149	ACTB
Role of IL-17A in Arthritis	0.609	0.0145	NFKB1
Myc Mediated Apoptosis Signaling	0.604	0.0143	YWHAZ
MSP-RON Signaling Pathway	0.593	0.0139	ACTB
Hypoxia Signaling in the Cardiovascular System	0.588	0.0137	LDHA
STAT3 Pathway	0.583	0.0135	FLT4
BMP signaling pathway	0.583	0.0135	NFKB1
Role of PI3K/AKT Signaling in the Pathogenesis of Influenza	0.573	0.0132	NFKB1
Angiopoietin Signaling	0.568	0.013	NFKB1
Signaling by Rho Family GTPases	0.564	0.00797	ACTB,NFKB1
CD40 Signaling	0.563	0.0128	NFKB1
IL-17A Signaling in Airway Cells	0.563	0.0128	NFKB1
Estrogen-Dependent Breast Cancer Signaling	0.559	0.0127	NFKB1
Regulation of IL-2 Expression in Activated and Anergic T Lymphocytes	0.559	0.0127	NFKB1
Role of BRCA1 in DNA Damage	0.554	0.0125	ACTB

Response			
Erythropoietin Signaling	0.549	0.0123	NFKB1
Altered T Cell and B Cell Signaling in Rheumatoid Arthritis	0.54	0.012	NFKB1
JAK/Stat Signaling	0.54	0.012	NFKB1
Growth Hormone Signaling	0.532	0.0118	A2M
Small Cell Lung Cancer Signaling	0.532	0.0118	NFKB1
HIPPO signaling	0.528	0.0116	YWHAZ
NF-κB Activation by Viruses	0.523	0.0115	NFKB1
PEDF Signaling	0.523	0.0115	NFKB1
Apoptosis Signaling	0.515	0.0112	NFKB1
IL-1 Signaling	0.503	0.0109	NFKB1
PPAR Signaling	0.503	0.0109	NFKB1
Ceramide Signaling	0.499	0.0108	NFKB1
Fcγ Receptor-mediated Phagocytosis in Macrophages and Monocytes	0.499	0.0108	ACTB
Prostate Cancer Signaling	0.492	0.0105	NFKB1
Sumoylation Pathway	0.488	0.0104	NFKB1
TR/RXR Activation	0.481	0.0102	ACACA
RANK Signaling in Osteoclasts	0.474	0.01	NFKB1
Antioxidant Action of Vitamin C	0.46	0.00962	NFKB1
Chronic Myeloid Leukemia Signaling	0.456	0.00952	NFKB1
Role of Macrophages,	0.455	0.0066	VCAM1,NFKB1

Fibroblasts and Endothelial Cells in Rheumatoid Arthritis			
IGF-1 Signaling	0.453	0.00943	YWHAZ
Type I Diabetes Mellitus Signaling	0.45	0.00935	NFKB1
Nitric Oxide Signaling in the Cardiovascular System	0.447	0.00926	FLT4
T Cell Receptor Signaling	0.447	0.00926	NFKB1
Glioma Signaling	0.431	0.00885	IDH1
HIF1α Signaling	0.422	0.00862	LDHA
Rac Signaling	0.422	0.00862	NFKB1
iCOS-iCOSL Signaling in T Helper Cells	0.416	0.00847	NFKB1
Pancreatic Adenocarcinoma Signaling	0.411	0.00833	NFKB1
Role of Tissue Factor in Cancer	0.411	0.00833	F2
NGF Signaling	0.411	0.00833	NFKB1
Renin-Angiotensin Signaling	0.408	0.00826	NFKB1
fMLP Signaling in Neutrophils	0.405	0.0082	NFKB1
Cellular Effects of Sildenafil (Viagra)	0.394	0.00794	ACTB
PI3K Signaling in B Lymphocytes	0.394	0.00794	NFKB1
CD28 Signaling in T Helper Cells	0.392	0.00787	NFKB1
14-3-3-mediated Signaling	0.384	0.00769	YWHAZ
Th1 Pathway	0.384	0.00769	NFKB1
P2Y Purigenic Receptor Signaling	0.376	0.00752	NFKB1

Pathway			
Corticotropin Releasing Hormone Signaling	0.372	0.00741	KRT1
Aryl Hydrocarbon Receptor Signaling	0.369	0.00735	NFKB1
Androgen Signaling	0.369	0.00735	NFKB1
Phagosome Maturation	0.364	0.00725	MPO
Hereditary Breast Cancer Signaling	0.357	0.00709	ACTB
Th2 Pathway	0.346	0.00685	NFKB1
Protein Kinase A Signaling	0.332	0.00521	YWHAZ,NFKB1
Relaxin Signaling	0.33	0.00649	NFKB1
PKCθ Signaling in T Lymphocytes	0.33	0.00649	NFKB1
Gαq Signaling	0.32	0.00629	NFKB1
GNRH Signaling	0.314	0.00617	NFKB1
eNOS Signaling	0.308	0.00606	FLT4
RhoGDI Signaling	0.289	0.00568	ACTB
Th1 and Th2 Activation Pathway	0.282	0.00556	NFKB1
Role of NFAT in Regulation of the Immune Response	0.28	0.00552	NFKB1
Dendritic Cell Maturation	0.274	0.00541	NFKB1
Regulation of the Epithelial-Mesenchymal Transition Pathway	0.271	0.00535	NFKB1
B Cell Receptor Signaling	0.269	0.00532	NFKB1
NRF2-mediated Oxidative Stress Response	0.266	0.00526	ACTB

Gap Junction Signaling	0.265	0.00524	ACTB
Axonal Guidance Signaling	0.264	0.00449	PFN1,NRP1
Calcium Signaling	0.255	0.00505	TPM4
ERK/MAPK Signaling	0.253	0.00503	YWHAZ
EIF2 Signaling	0.236	0.00472	ACTB
Systemic Lupus Erythematosus Signaling	0.229	0.00461	C8A
Role of Osteoblasts, Osteoclasts and Chondrocytes in Rheumatoid Arthritis	0.216	0.00439	NFKB1
Phospholipase C Signaling	0.208	0.00426	NFKB1
Opioid Signaling Pathway	0.206	0.00422	NFKB1
Colorectal Cancer Metastasis Signaling	0.199	0.00412	NFKB1

Table S6. The highest scoring IPA network among the differentially expressed proteins

Symbol	Entrez Gene Name	UniProt Accession	Expr Log Ratio	Location	Family	Biomarker Application(s)
A2M	alpha-2-macroglobulin	P01023	-1.48211	Extracellular Space	transporter	
APOA1	apolipoprotein A1	P02647	-1.72634	Extracellular Space	transporter	diagnosis, efficacy, unspecified application
APOA2	apolipoprotein A2	P02652	-1.43576	Extracellular Space	transporter	efficacy
APOC2	apolipoprotein C2	P02655	-0.79234	Extracellular Space	transporter	efficacy
APOC3	apolipoprotein C3	P02656	-0.71175	Extracellular Space	transporter	diagnosis, efficacy
APOD	apolipoprotein D	P05090	-1.54837	Extracellular Space	transporter	safety
APOF	apolipoprotein F	Q13790	-0.81114	Extracellular Space	transporter	
APOL1	apolipoprotein L1	O14791	-0.63721	Extracellular Space	transporter	
CFHR1	complement factor H related 1	Q03591	0.605943	Extracellular Space	other	
CRP	C-reactive protein	P02741	1.143304	Extracellular Space	other	diagnosis, disease progression, efficacy, prognosis, safety, unspecified application
DBH	dopamine beta-hydroxylase	P09172	-0.6882	Cytoplasm	enzyme	
F2	coagulation factor II, thrombin	P00734	1.046538	Extracellular Space	peptidase	diagnosis, unspecified application
FCN2	ficolin 2	Q15485	-0.6872	Extracellular Space	peptidase	efficacy
Fibrinogen	--	--	--	Plasma Membrane	complex	diagnosis, disease progression, efficacy, prognosis, safety
IL1	--	--	--	Extracellular Space	group	diagnosis, efficacy, prognosis, response to therapy
Jnk	--	--	--	Cytoplasm	group	
LBP	lipopolysaccharide binding protein	P18428	1.150616	Plasma Membrane	transporter	diagnosis, efficacy
LDL	--	--	--	Plasma Membrane	complex	efficacy, unspecified application
LTF	lactotransferrin	P02788	0.800676	Extracellular Space	peptidase	efficacy, unspecified application
MPO	myeloperoxidase	P05164	1.428841	Cytoplasm	enzyme	diagnosis, efficacy, unspecified application
NFkB (complex)	--	--	--	Nucleus	complex	diagnosis, disease progression, efficacy, prognosis
NFKB1	nuclear factor kappa B subunit 1	P19838	-1.07519	Nucleus	transcription regulator	
P38 MAPK	--	--	--	Cytoplasm	group	

S100A8	S100 calcium binding protein A8	P05109	0.938674	Cytoplasm	other	diagnosis, efficacy, unspecified application
SAA1	serum amyloid A1	P0DJI8	-1.65251	Extracellular Space	transporter	diagnosis, unspecified application
SERPINA1	serpin family A member 1	P01009	-1.72803	Extracellular Space	other	diagnosis, unspecified application
SERPINA5	serpin family A member 5	P05154	-2.17098	Extracellular Space	other	diagnosis
SRC (family)	--	--	--	Cytoplasm	group	
TCF	--	--	--	Other	group	
TCR	--	--	--	Plasma Membrane	complex	
TF	transferrin	P02787	-1.59683	Extracellular Space	transporter	efficacy, prognosis, safety, unspecified application
TFRC	transferrin receptor	P02786	-0.81163	Plasma Membrane	transporter	diagnosis, efficacy
VCAM1	vascular cell adhesion molecule 1	P19320	-0.6009	Plasma Membrane	transmembrane receptor	diagnosis, disease progression, efficacy, prognosis, unspecified application
VCL	vinculin	P18206	1.685397	Plasma Membrane	enzyme	unspecified application
VEGF	--	--	--	Extracellular Space	group	diagnosis, disease progression, efficacy, prognosis, response to therapy, unspecified application

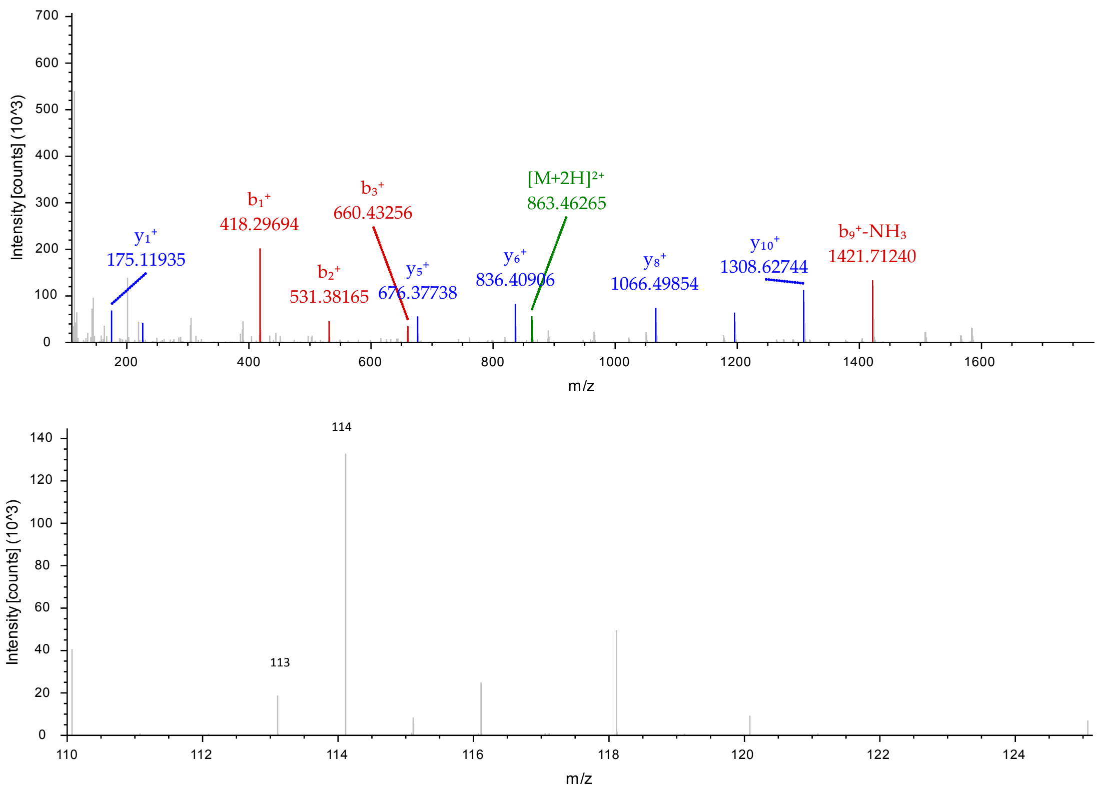

Fig. S3 Examples of MS/MS spectra of a 2.01-fold up-regulated protein—S100-A8, illustrating the degree of relative quantitative measurement is consistency. Healthy control and PQ poisoning patients were labeled with 113- and 114- reporter isobaric reagent respectively. The MS/MS spectra of a peptide- LLETECPQYIR with m/z of 863.46 Da (z = 2), related b ion and y ion were shown.

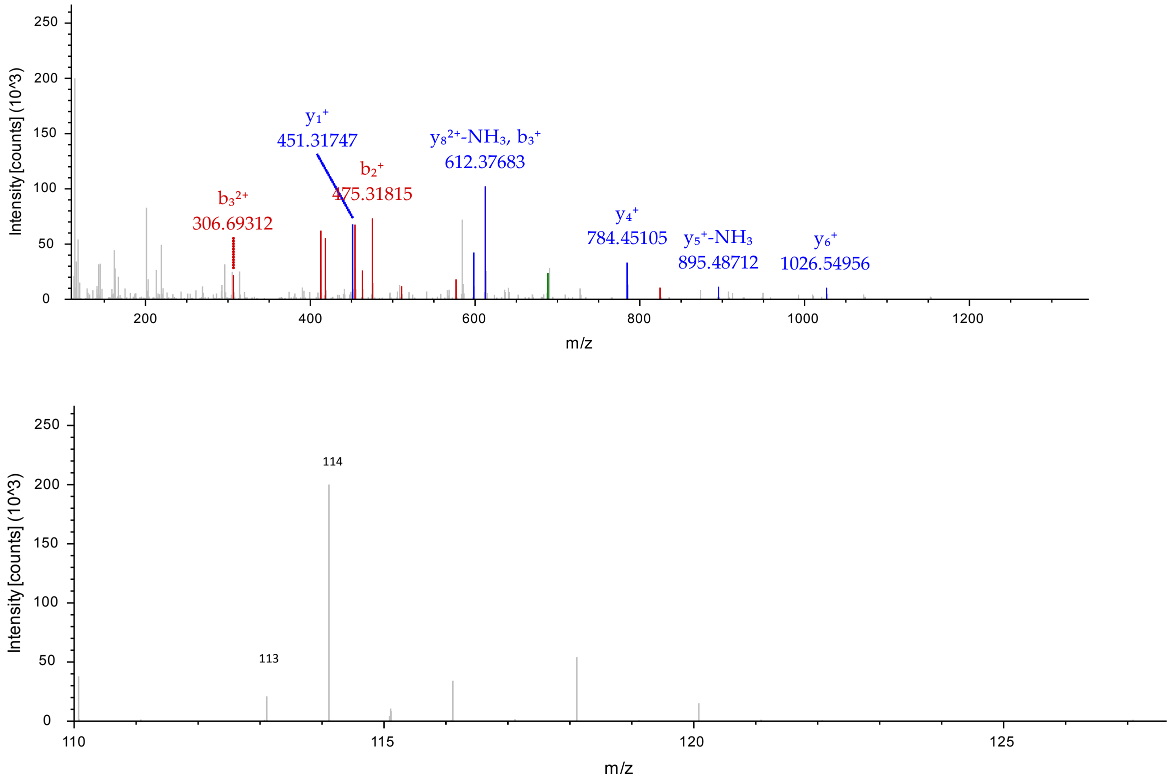

Fig. S4 Examples of MS/MS spectra of a 2.25-fold up-regulated protein—S100-A9, illustrating the degree of relative quantitative measurement is consistency. Healthy control and PQ poisoning patients were labeled with 113- and 114- reporter isobaric reagent respectively. The MS/MS spectra of a peptide- LTWASHEK with m/z of 527.30 Da Da (z = 3), related b ion and y ion were shown.

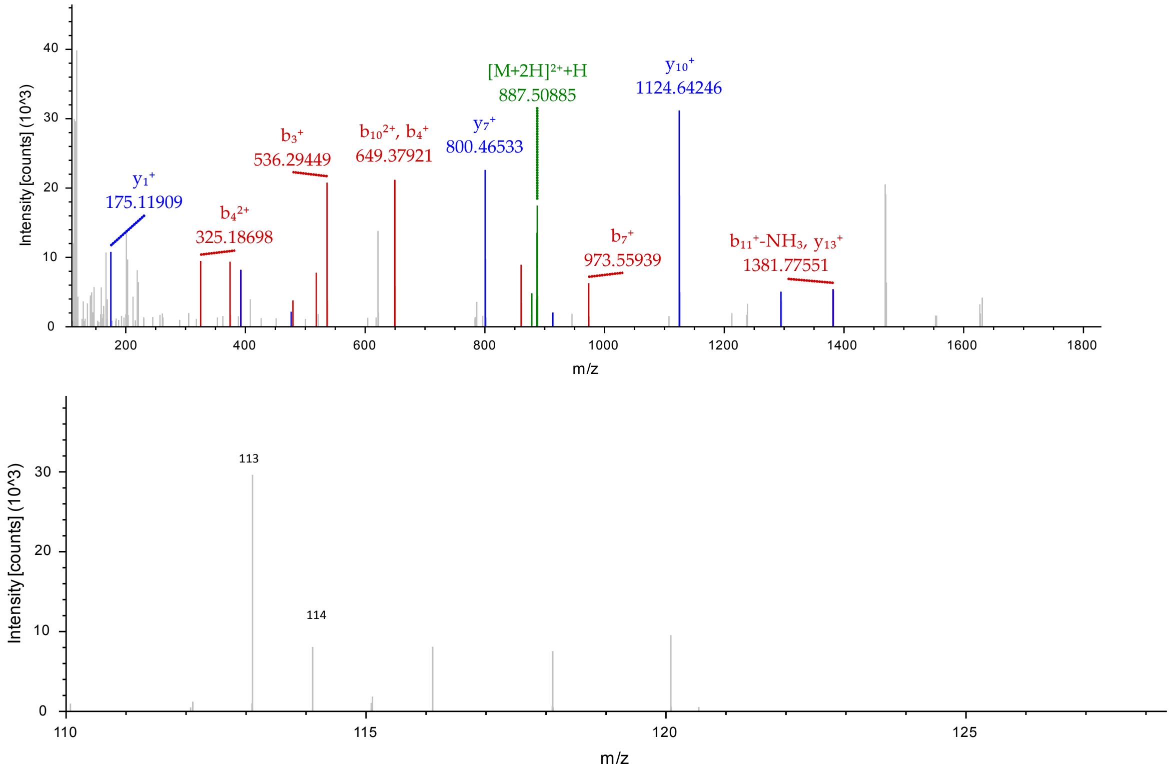

Fig. S5 Examples of MS/MS spectra of a 0.52-fold up-regulated protein—TfR1, illustrating the degree of relative quantitative measurement is consistency. Healthy control and PQ poisoning patients were labeled with 113- and 114- reporter isobaric reagent respectively. The MS/MS spectra of a peptide- SSGLPNIPVQTISR with m/z of 887.01 Da Da (z = 2), related b ion and y ion were shown.

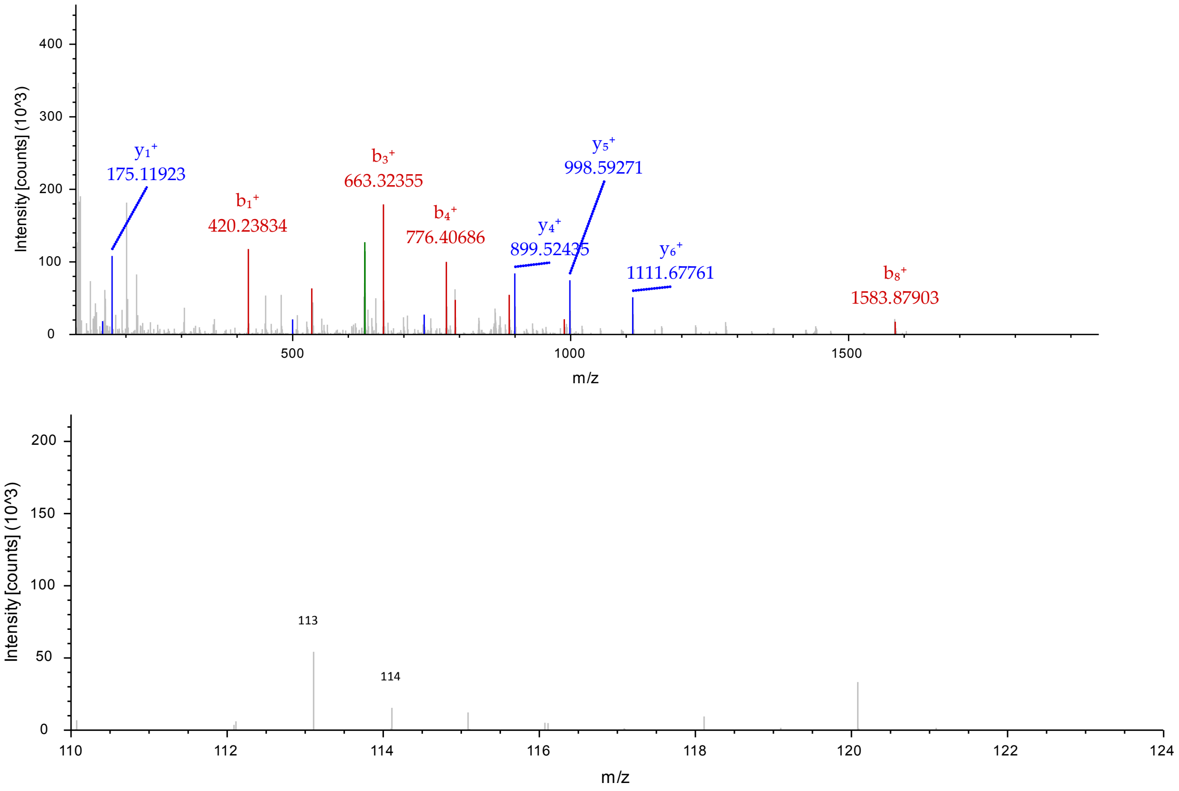

Fig. S6 Examples of MS/MS spectra of a 0.51-fold up-regulated protein—SAP, illustrating the degree of relative quantitative measurement is consistency. Healthy control and PQ poisoning patients were labeled with 113- and 114- reporter isobaric reagent respectively. The MS/MS spectra of a peptide- DNELLVYKER with m/z of 629.69 Da Da (z = 3), related b ion and y ion were shown.

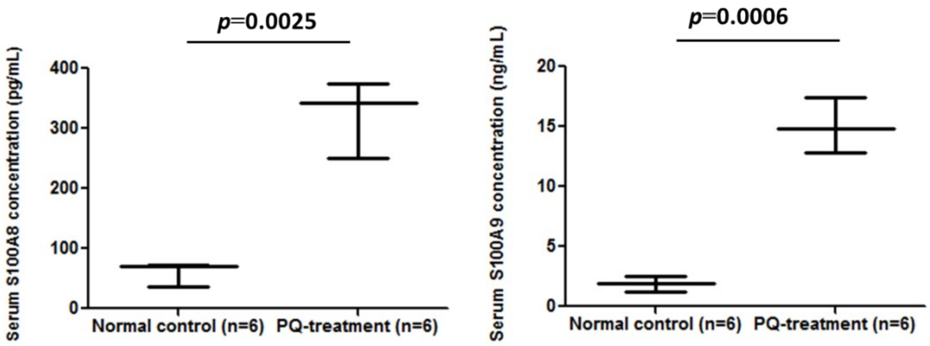

Fig. S7 Serum levels of S100A8 and S100A9 in murine mode (n=6) with normal control and PQ-treatment, respectively. (a) serum S100A8 is increased in PQ-treatment murine models compared with normal controls (normal 59.6±11.7 pg/mL versus PQ-treatment 321.7±37.2 pg/mL, *p*=0.0025). (b) serum S100A9 is increased in PQ-treatment murine models compared with normal controls (normal 1.88±0.37 ng/mL versus PQ-treatment 15.0±1.31 ng/mL, *p*=0.0006).

$$PQ + e^{-} \rightarrow PQ^{*}$$
$$PQ^{*} + O_2 \rightarrow PQ + O_2^{*-}$$
$$O_2^{*-} + O_2^{*-} + H^{+} \rightarrow O_2 + H_2O_2$$

Fig. S8 Proposed mechanism for H_2O_2 production by paraquat (PQ) (*cited from J Biol Chem 282, 14186-14193 (2007)*).
